# Interplay of the Bacterial Ribosomal A-Site, S12 Protein Mutations and Paromomycin Binding: A Molecular Dynamics Study

**DOI:** 10.1371/journal.pone.0111811

**Published:** 2014-11-07

**Authors:** Joanna Panecka, Cameron Mura, Joanna Trylska

**Affiliations:** 1 Division of Biophysics, Institute of Experimental Physics, University of Warsaw, Warsaw, Poland; 2 Interdisciplinary Centre for Mathematical and Computational Modelling, University of Warsaw, Warsaw, Poland; 3 Department of Chemistry, University of Virginia, Charlottesville, VA, United States of America; 4 Centre of New Technologies, University of Warsaw, Warsaw, Poland; University of Lethbridge, Canada

## Abstract

The conformational properties of the aminoacyl-tRNA binding site (A-site), and its surroundings in the *Escherichia coli* 30S ribosomal subunit, are of great relevance in designing antibacterial agents. The 30S subunit A-site is near ribosomal protein S12, which neighbors helices h27 and H69; this latter helix, of the 50S subunit, is a functionally important component of an intersubunit bridge. Experimental work has shown that specific point mutations in S12 (K42A, R53A) yield hyper-accurate ribosomes, which in turn confers resistance to the antibiotic ‘paromomycin’ (even when this aminoglycoside is bound to the A-site). Suspecting that these effects can be elucidated in terms of the local atomic interactions and detailed dynamics of this region of the bacterial ribosome, we have used molecular dynamics simulations to explore the motion of a fragment of the *E. coli* ribosome, including the A-site. We found that the ribosomal regions surrounding the A-site modify the conformational space of the flexible A-site adenines 1492/93. Specifically, we found that A-site mobility is affected by stacking interactions between adenines A1493 and A1913, and by contacts between A1492 and a flexible side-chain (K43) from the S12 protein. In addition, our simulations reveal possible indirect pathways by which the R53A and K42A mutations in S12 are coupled to the dynamical properties of the A-site. Our work extends what is known about the atomistic dynamics of the A-site, and suggests possible links between the biological effects of hyper-accurate mutations in the S12 protein and conformational properties of the ribosome; the implications for S12 dynamics help elucidate how the miscoding effects of paromomycin may be evaded in antibiotic-resistant mutants of the bacterial ribosome.

## Introduction

**Figure 1:**
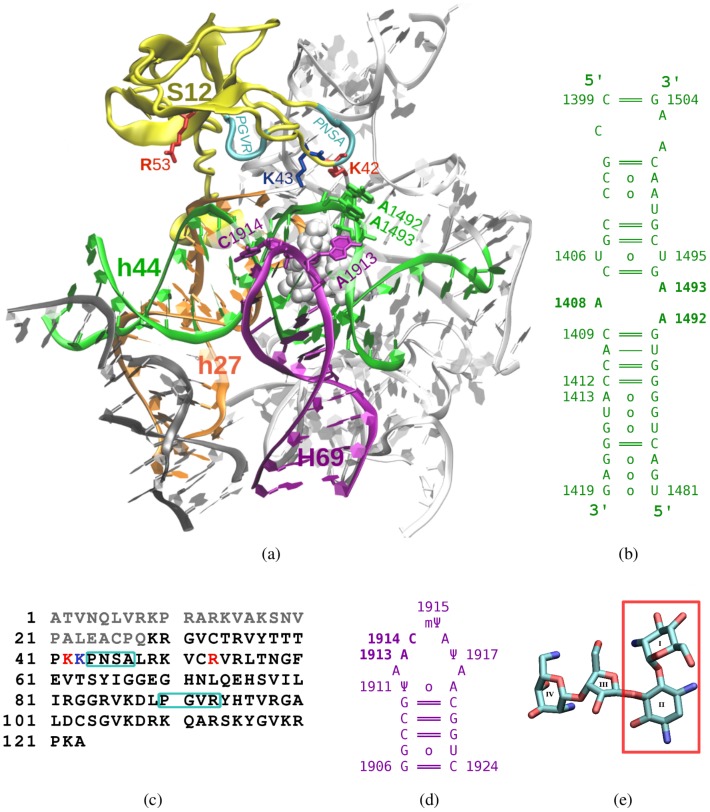
a) A fragment of the *E. coli* 30S ribosomal subunit is shown, including part of the h44 helix with the A-site (green), protein S12 (gold), helices H69 (purple) and h27 (orange), the remaining 16S rRNA (light grey) and paromomycin (white vdW spheres). b) Secondary structure scheme of the h44 helix fragment included in our MD system. c) The S12 sequence, indicating amino acids in the globular “head” (black) and “tail” regions (grey), and two conserved sequence motifs (light blue frames); mutations examined in this work are in red, and the K43 amino acid (see text) is dark blue. d) Secondary structure scheme of the H69 hairpin. e) The covalent structure of paromomycin (in non-hydrogen representation), showing the neamine core (red box).

Ribosomes translate messenger RNAs (mRNAs) into proteins with high fidelity, and in bacteria the small (30S) subunit of the ribosome helps control translational fidelity [1]. During the elongation phase of translation, the anticodon of an incoming aminoacylated transfer RNA (tRNA) binds to the mRNA codon at a specific site on the 30S subunit, known as the aminoacyl-tRNA binding site (A-site). A key event is molecular recognition of cognate tRNA and rejection of incorrectly charged (near-cognate) tRNAs, thus ensuring that the correct amino acid is appended to the elongating peptide [2]. The most important ribosomal nucleotides in this process are two adenines, 1492 and 1493 (according to the standard *E. coli* ribosomal RNA sequence numbering), located in helix h44 (Fig 1a). The mobility of these adenines is crucial for proper codon–anticodon interactions [3], [4]: To enable binding of the tRNA and its anticodon triplet, the two adenines flip out of a bulge through the minor groove [5]. The adenines exist in a dynamic equilibrium between *flipped-in* and *flipped-out* conformations [6], and the latter state favors complexation with the tRNA anticodon [7]. This process underlies the mechanisms of action of an entire class of A-site–binding aminoglycosidic antibiotics [8], which lock the two adenines in extra-helical conformations [9], [10] and thereby promote the incorporation of near-cognate tRNAs. Such mis-incorporation yields dysfunctional proteins and, ultimately, bacterial cell death. The A-site adenines are also responsible for relaying signals to the large (50S) subunit and for interactions between the 30S and 50S subunits, as part of a ‘B2a bridge’. Therefore, the A-site nucleotides function in a structurally intricate ribosomal context, in close vicinity to ribosomal components such as the S12 protein and helix H69 of the 50S subunit (Fig 1a). The steric congestion and intermolecular contacts in this region of the ribosome suggest that the dynamics of the A-site and its ribosomal surroundings modulate one another.

The ribosomal protein S12, located proximal to the A-site (Fig 1a), is a key contributor to the fidelity of mRNA decoding [11]. S12 is one of few 30S proteins located near the interface with the 50S ribosomal subunit [12]. The extended N-terminal tail of S12 is anchored in the interior of the small subunit and its globular domain is located on the surface. The S12 sequence is highly conserved in bacteria, and there are sequence elements that are universally conserved [13], such as the PNSA (residues 44–47) and PGVR (90–93) motifs (Fig 1c). Interestingly, the PNSA motif is located near the key adenines A1492 and A1493. The S12 protein has been found to be important in ensuring translational accuracy, and is critical for proper ribosomal function [5], [14]. Experimental studies of S12 in bacterial and eukaryotic species, including the yeast *S. cerevisiae*, have shown that many mutations in the S12-encoding gene (*rpsL*) yield error-restrictive (‘hyper-accurate’) translation [15], [16], [17]. Also, *rpsL* mutations were found to be the main determinants in bacterial resistance to the aminoglycosidic antibiotic streptomycin [18], [19], which binds in the vicinity of the A-site and causes translational misreading. This misreading effect likely results from antibiotic-induced distortions in the A-site [20]; notably, these effects can be silenced by hyper-accurate mutations in S12 [11].

Intriguingly, some of the known S12 mutations also confer resistance to the aminoglycoside paromomycin (PAR) [17], which binds directly in the A-site and does *not* contact the S12 protein. In the present study, we have focused on S12 mutations at two positions: K42 and R53. In *E. coli*, the two ‘hyper-accurate’ S12 mutants, K42A and R53A, have been shown to confer significant resistance to micromolar concentrations of PAR [17]. In the yeast homolog of S12, termed ‘S28’, substitution at position K62 (homologous to *E. coli* K42) by R, N, T or Q decreases the PAR miscoding effects in cell-free translation studies with the mutant ribosomes [15]. The other mutation, R53A, suppresses the miscoding effect of this antibiotic [17], despite being quite distant from the PAR binding site (Fig 1a). Residue R53 is likely of great structural and functional importance, as it is conserved in a wide range of bacteria and eukaryotes (e.g. *Homo sapiens*, *Mus musculus*, *Rattus norvegicus*). Of all S12 amino acids outside the PNSA motif, R53 is located the closest to ribosomal RNA (rRNA) helices 44 (h44) and 27 (h27). Experiments have shown that mutations at the h44–h27–S12 junction affect the proofreading step of mRNA decoding [14]. Taken together, these experimental results show that sequence variation in S12 can weaken the miscoding effect of 2-deoxystreptamine aminoglycosides that bind at the A-site bulge, even though these mutations are not *directly* involved in the binding mechanism of these aminoglycosides. Because the effect is *not* directly chemical (say, for instance, alteration of a chemical moiety involved in catalysis), it must be dynamical, possibly stemming from mutation-induced conformational changes. Molecular dynamics (MD) simulations afford a method to study such phenomena.

Helix 69 (H69) is another functionally important element that lies near the A-site in intact ribosomes. H69 is part of the large, 50S subunit (Fig 1ad); it contacts h44 and forms the B2a intersubunit bridge, which is an important element of the translational machinery [21]. Nucleotides from the H69 loop region are in close contact with h44. For instance, A1912 (H69 [50S]) interacts with the G1494:C1407 pair (h44 [30S]), and A1919 (H69 [50S]) stabilizes the U1406:U1495 pair (h44 [30S]) which, in turn, interacts with aminoglycosides in the A-site [22]. H69 also includes modified ribonucleotides in the native ribosome: two pseudouridines (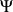
1911 and 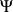
1917) and a methylated 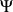
1915 are important for H69 stability and function [23], [24]. Experiments have demonstrated that even though deletion of H69 is lethal *in vivo* such deletion does not affect translational accuracy *in vitro* [25]. However, other experimental data show that, for instance, the m
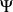
1915A mutation in H69 decreases translational accuracy [26], possibly via interactions with the D-stem of A/T-site tRNA rather than with the decoding center. However, in the crystal structures of the ribosome, e.g. [27] or [28], we observe contacts between nucleotides of the decoding site and H69 (A1913, C1914). An interesting question is the degree to which interatomic contacts between H69 nucleotides and core elements of the decoding center (e.g. A1492, A1493) modulate the decoding process (and its antibiotic sensitivity).

A few large-scale computational studies have examined global ribosome motions during the translation process [29], [30], [31], [32], [33], [34], [35]. Also, atomic details of the decoding mechanism and the mobility of A-site A1492 and A1493 have been computationally examined [6], [10], [22], [36], [37], [38], [39], [40], [41], [42]. However, there have been no theoretical studies of S12 protein structure/function relationships, particularly as regards its specific amino acid sequence, the decoding mechanism, and the molecular determinants of antibiotic binding. Also, the dynamics of the H69 hairpin and its influence on A-site dynamics remains relatively unstudied, despite the proximal location of H69 to the mRNA decoding site. These gaps in knowledge motivated us to explore the dynamical effects of amino acid substitutions in S12 and the influence of H69–A-site interactions on the dynamics of the key A-site adenines.

Here, we report our characterization of the dynamics of a ribosomal region containing the A-site, S12 protein, and rRNA helices H69 and h44 (Fig 1a). Our systems include the wild-type S12 protein and its K42A and R53A variants; also, each system was simulated either with or without PAR in order to analyze the link between S12 mutations and the antibacterial action of this aminoglycoside. We compared the dynamics of these systems to find possible connections between local conformational changes and the overall translational hyper-accuracy that has been experimentally characterized for these S12 mutants [17]. We also simulated a bare helix h44 fragment, enabling us to assess how the flexibilities of h44 nucleotides are coupled to the placement of this helix within the structural context of an intact ribosome. To our knowledge, these are the first MD studies of the dynamical coupling between the ribosomal A-site, the S12 protein variants, and the surrounding rRNA elements (e.g. helices H69 and h44).

## Methods

### System selection & setup

The initial coordinates were taken from a crystallographic structure of the *E. coli* ribosome bound to neomycin, including both the 30S (pdb 2QAL) and 50S (pdb 2QAM) subunits [27]. As of March 2010, this was the highest resolution structure available (3.21 Å) among the *E. coli* systems containing an aminoglycoside at the A-site; our simulation system was drawn from the *E. coli* ribosome without mRNA and tRNA, as we found no structure with sufficiently long mRNA fragments (extending to both sides of the A-site region). We began by excising a fragment of the ribosome comprised of all protein and RNA residues within 30 Å of the A-site antibiotic. In so doing, only intact residues and discrete elements of rRNA (i.e. full nucleotides) were included in the simulation systems. The final system, shown in Fig 1a, consisted of 239 nucleotides and 98 amino acids: (i) 16S rRNA, including part of helix h44 (nts 10
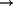
25, 516
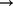
533, 559
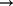
577, 786
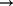
796, 812
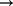
819, 882
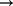
927, 1392
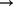
1419, 1481
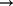
1531); (ii) 23S rRNA with helix H69 (1906
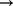
1924, 1930
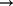
1936, 1945
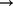
1950, 1960
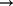
1969); (iii) most of the S12 protein (amino acids 1
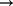
98); and (iv) in specific cases, the antibiotic PAR bound to the A-site of the 30S subunit.

The neomycin molecule in the starting crystal structure was replaced by PAR from an A-site model (pdb 1J7T [43]) by superimposing heavy atoms of the neamine core of both structures (see ring systems in Fig 1e). These antibiotics differ by only a single substituent – an amino group in neomycin is a hydroxyl in PAR. All hydrogens in PAR were energy-minimized, i.e. subjected to 8000 steps of steepest descent, followed by 2000 steps of conjugate gradient potential energy minimization. For simulation systems without antibiotic, neomycin was removed and the above energy minimization strategy was applied to nucleotides in the A-site region (nts 1404
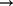
1412 and 1488
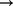
1497 of 16S RNA).

Note that we removed the second neomycin bound to the H69 loop in the original crystal structure [27]. This was done because PAR has an order of magnitude lower affinity towards H69 than does neomycin [44]. Also, the conformation of H69 is similar in all bacterial ribosomal structures irrespective of the presence of antibiotic; relatively large conformational changes in H69 occur only during the recycling or termination stage of translation [27]. Finally, because pseudouridylation in helix H69 may be important for ribosome function [23], [24], [26], our MD systems included all known chemical modifications to the ribonucleotides [45].

### A restrained fragment approach

Atomically detailed MD simulations of the entire ribosome are possible, in principle [46], [47], but such calculations are extremely computationally expensive. Instead, our study focuses on the relatively local dynamics of a small, well-defined region of the ribosome, obviating the need for computationally demanding simulations of the entire ribosome. To render our systems tractable without completely neglecting the ribosomal surroundings, we applied a ‘restrained fragment’ approach. We began by excising specific (contiguous) fragments of the ribosome, and then restraining particular parts of the system so as to mimic the constraints implicitly imposed by the surrounding 30S environment. First, weak positional restraints (0.35 kcal/mol/Å^2^) were applied to atoms in each terminal residue – specifically, the O5′ of each 5′ terminal nucleotide, O3′ of 3′ termini, and the C-terminal oxygen and N-terminal nitrogen of S12. Additionally, any other given residue, 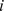, was restrained based on the number of contacts between 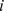 and other atoms, 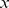, in the remainder of the excised ribosomal structure, for distances 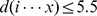
Å. Specifically, harmonic force constants were scaled linearly in a range from 0 (if no contacts) 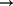 0.35 kcal/mol/Å^2^ (if 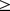
20 contacts). Notably, our spatial restraint scheme did not result in restraints being applied to any of the residues of interest that were analyzed in detail in this work (e.g., K42).

For residues in our excised systems that contact other residues in the 30S subunit, application of this restraint scheme helps mimic the local steric environment in the native ribosome. A benefit of our approach is that we can simulate the A-site in atomistic detail and in an integrated manner (accounting for the surrounding ribosome) with relatively little computational cost (thus allowing longer sampling times). Reasonably low deviations from the initial crystal structure (see root-mean-square deviations [RMSDs] in Table S1 in File S1) demonstrate the structural stability of MD simulation systems restrained in this way. A limitation of our approach is that, for instance, global opening/closing of the 30S subunit [48] cannot be captured in our simulations. Though this ‘restrained fragment’ approach limits us to only local motions near the A-site, it does model the network of interatomic contacts that define the simulated fragment in the context of its ribosomal surroundings.

In simulating helix h44 in isolation and without PAR, we included the 30S nucleotides 1404
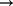
1419 and 1481
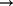
1497 (for sequence see Fig 1b). Here, positional harmonic restraints were applied to only the heavy atoms of terminal residues, in order to mimic the noncovalent interactions with flanking h44 nucleotides that were not explicitly included.

Our simulation study is not free of limitations. For example, the restrained fragment approach, described above, is a balance between (*i*) physical realism (we restrict our system to a subset of the ribosome that interests us) and (*ii*) the benefits of greater computational efficiency, such as the more extensive sampling afforded by restricted system size. Our simulations do not capture global dynamics of the ribosome, such as the intersubunit rotations and L1 stalk motion very recently reported by Bock et al. [32]. Such movements are functionally important and could alter the configurations of h44 and S12, but they lie beyond the scope of this study. Our present study aims to investigate – in detail – the local motions of the ribosome and, indeed, we did detect certain local effects that are consistent with experimental data.

### Force-field usage

Paromomycin (Fig 1e) was parameterized with the generalized Amber force-field (GAFF) [49]; its partial charges were calculated in Antechamber (AmberTools1.3 package [50]) with the AM1-BCC [51] semi-empirical quantum method, which is known to reproduce well HF
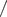
6-31G* RESP [52] charges. The glycosidic
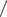
aglycosidic dihedral angles and inter-proton distances in PAR, as derived from the current MD study versus NMR data [53], have been compared elsewhere [54]. Unless otherwise noted, RNA and protein were treated with the Amber parm99 [52] and parm99SB [55] force-fields, respectively (RNA force-field choice is discussed in detail in Section S1 of File S1). Parameters for modified rRNA residues were taken from Aduri et al. [56]. The TIP3P water model [57] was used for explicit solvent, along with standard Amber Lennard-Jones parameters for Na^+^ (radius = 1.868 Å, well depth = 0.00277 kcal/mol) [58] and Cl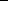 (2.47 Å, 0.1 kcal/mol) [59] ions.

### Molecular dynamics calculations and protocols

Each S12 simulation system was immersed in a truncated octahedral box of explicit solvent, with a minimal clearance of 12 Å for each atom from the cell faces. The negatively charged systems were neutralized by adding Na^+^ ions in place of water molecules at positions of local minima of the electrostatic potential (tleap module of AmberTools 1.3 [50]). Next, equal numbers of Na^+^ and Cl^−^ ions were added at random positions to yield an ionic strength of 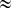
0.1 M. Each of the final simulation systems contained over 60 000 atoms, including roughly 17 500 water molecules.

MD simulations were performed using NAMD (ver. 2.6/2.7) [60] in the NpT ensemble, with a constant pressure of 1 bar regulated by the Langevin piston method [61] and a constant temperature of 310 K ensured via the Langevin thermostat. The SHAKE algorithm [62] was used to constrain H–X bonds, thereby permitting a 2-fs integration timestep. Periodic boundary conditions (PBC) were applied instead of, for instance, spherical (stochastic) boundary conditions [42]. We employed truncated octahedral cells; this PBC geometry, which has become standard in atomistic MD simulations, substantially reduces the simulation system size, versus rectangular or cubic PBC cells. No problems were detected as to our choice of PBC method. The possibly restriced mobility of some nucleotides and amino acids, lying near the spatial boundary of the excised ribosome environment, was accounted for via aforementioned restrained fragment approach. Long-range interactions were treated via the particle-mesh Ewald method [63], with a grid spacing of about 1.0
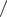
Å; a 10 Å spherical cutoff was used in evaluating non-bonded interactions.

Our MD equilibration protocol, specifically tailored to nucleic acids [64], followed the approach in our recent simulations of modified oligonucleotides [65]. Specifically, thermalization stages were performed in the NVT ensemble and consisted of two substages. During the first 65 ps, the temperature was linearly ramped from 30 to 310 K with harmonic restraints of 50 kcal/mol/Å^2^ imposed on solute heavy atoms. Spring constants were then relaxed to 25 kcal/mol/Å^2^, and the simulation continued for 35 ps. Next, 300 ps of equilibration was performed in the NpT ensemble, with restraints gradually decreased from 5 kcal/mol/Å^2^ to nearly 0 by halving the force constant every 50 ps. The final equilibration phase (NpT) lasted 600 ps (bringing the total to 1 ns), and was followed by 50 ns of the production-stage dynamics.

For each of the six S12 systems that were simulated, three independent 50-ns trajectories were computed from the same starting structure but with different initial velocities, yielding a total sampling time of 0.9 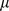
s. Thus, our simulation systems were extensively sampled on the ns
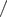
sub-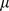
s timescale. This clearly precludes the possibility of converged microscopic conformational properties on the 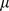
s timescale for the most flexible modes of nucleotides A1492 and A1493 (see 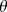 pseudo-dihedral angles in Fig S1 in File S1). To quantify this effect, we performed a clustering analysis. Conformational clustering of mutual A1492 and A1493 configurations in the three MD trajectories reveals unequal cluster populations in the three MD trajectories; see, for instance, the data for the WT systems shown in Fig S2e in File S1. Indeed, rare events, such as flipping of the A1492 and A1493 bases, may not be well-sampled even in a 1-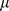
s classical, equilibrium MD simulation, as seen in our recent study of bacterial and human ribosomal A-site models [66]. Regardless, computed average RMS fluctuations as a function of time confirm the stability and convergence of the dynamics of the chosen subsystems (H69, S12, h44) on the timescale of greatest interest in the present work (see Fig S2b–d in File S1). Though full equilibration of our systems on longer timescales is not assured, our aim was to examine local conformational changes (near the A-site) that occur in similar regions of the free energy surface as the starting crystal structure. Our goal was *not* to examine the full mRNA decoding process, which would require at least 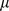
s-long simulations of the entire ribosome in order to account for global ribosomal rearrangements (which also involve mRNAs and tRNAs). In studying the dynamical stability of our systems near the crystallographic starting conformation, even a 10-ns timescale was found to be sufficient for stabilization of structural/geometric observables.

### Trajectory analysis

In most calculations (those involving comparisons of the S12 systems), analyses were applied to the last 40 ns of the production phase of each trajectory. The largest changes in backbone RMSD, versus initial structures, typically occurred during the first 10 ns of each trajectory (Fig S2a in File S1). The mean RMSDs calculated for the remaining 40 ns of each trajectory lay between 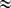
1.4–1.6 Å, with standard deviations near 0.1 Å (Table S1 in File S1). Each system appears to be equilibrated during this stage of the simulation. The overall stabilities of the important fragments in the simulated systems are also discussed in the Supporting Information (Sections S2 and S3 in File S1); RMSDs and representative conformations for these regions are in Fig S3 in File S1.

To obtain families of representative conformations for A1492 and A1493, we used the 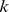-means clustering algorithm (*kclust*), implemented in the MMTSB suite [67]. This clustering method partitions configurational space into distinct subspaces according to a predefined RMSD value; therefore, the resultant number of clusters reflects the conformational variability of a given structural fragment. The optimal cluster radius was chosen based on visual inspection of the trajectories, and was found to be 3.5 Å. Prior to the actual clustering calculation, trajectory frames were superimposed based on heavy atoms of the helix h44 fragment.

Base stacking was estimated from the distance between the centers of nucleobases, with stacking assumed for distances less than 5.5 Å. Hydrogen bonds (H-bonds) were analyzed with the *ptraj* program of AmberTools1.3. Our H-bond analyses used a 3.5 Å cutoff for donor
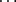
acceptor distances and a 120°C cutoff for the donor—proton
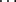
acceptor angle. Simulation-derived quantities (root-mean-square fluctuations [RMSFs], root-mean-square deviations [RMSDs], occupancies, etc.) were further averaged over three independent trajectories for each MD system variant, unless otherwise noted.

To characterize the conformations of A1492 and A1493, we used a center-of-mass pseudo-dihedral angle (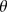), introduced in [68] and shown in Fig S1a in File S1. For A1492, 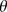 is defined by (*i*) the center of the neighboring base pair (C1407:G1491), (*ii*) the neighboring sugar (G1491), (*iii*) the A1492 sugar, and (*iv*) the A1492 nucleobase (and analogously for A1493). Values near 0° correspond to *flipped-in* configurations of the nucleobases, while the fully *flipped-out* states are characterized by 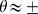
180°. Similarly as in ref. [69], throughout the text, we assumed that A1492 is flipped-in with 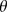 in range [−45°, 50°] and A1493: [−45°, 40°]. For instance, in Fig S1a in File S1 A1492 is in a *flipped-out* state, while A1493 is *flipped-in*. Glycosidic angles were also analyzed, with the following definitions of ranges: (*i*) *anti* = [−200°, −110°], (*ii*) high-*anti* = [−110°, −20°], (*iii*) *syn* = [20°, 80°]. The distributions of various dihedral angles and distances are plotted as smoothed histograms with bin-widths of 10° or 0.5 Å, respectively.

Molecular structures were illustrated in VMD 1.9 [70]. Numerical data were processed with Matlab 7.12.0 (R2011a) (http://www.mathworks.com) and graphical plots were created using Gnuplot 4.6 (http://www.gnuplot.info).

### Sequences and nomenclature

For comparison with other bacterial species, S12 protein sequences were analyzed and compared using the UniProt database and search engine (http://www.uniprot.org, accessed 21 Jun 2013). Only curated sequences of Proteobacteria were used, and the sequences were aligned using the FSA software [71].

Fragments of our simulation system (Fig 1) are named as per the standard *E. coli* numbering scheme: (i) h44 – nucleotides 1399–1419 and 1481–1504, (ii) H69 – nucleotides 1906–1924, (iii) S12 “head” – amino acids 29–98, (iv) S12 “tail” – amino acids 1–28. RNA and protein atom names used in the text are standard names from the Amber force-field.

The simulation systems that include S12 are referred to as “WT”, “K42A” and “R53A” for wild-type protein and the two mutants, respectively. The “*PAR*” subscript further denotes that paromomycin was included in the system. For example, a simulation system containing the K42A mutant of S12, with PAR bound, would be labeled “K42A
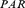
”.

## Results and Discussion

We present results of MD simulations of a ribosomal region (Fig 1a) containing the *wild-type* (native) S12 protein, as well as its K42A and R53A point mutants (see protein sequence in Fig 1c). Our analyses focus primarily on the dynamics of two key adenines in the A-site (A1492 and A1493, from rRNA helix h44), and on their interactions with the S12 protein and the H69 rRNA helix.

### Local mobility of A1492 and A1493 in the A-site, without paromomycin

**Figure 2:**
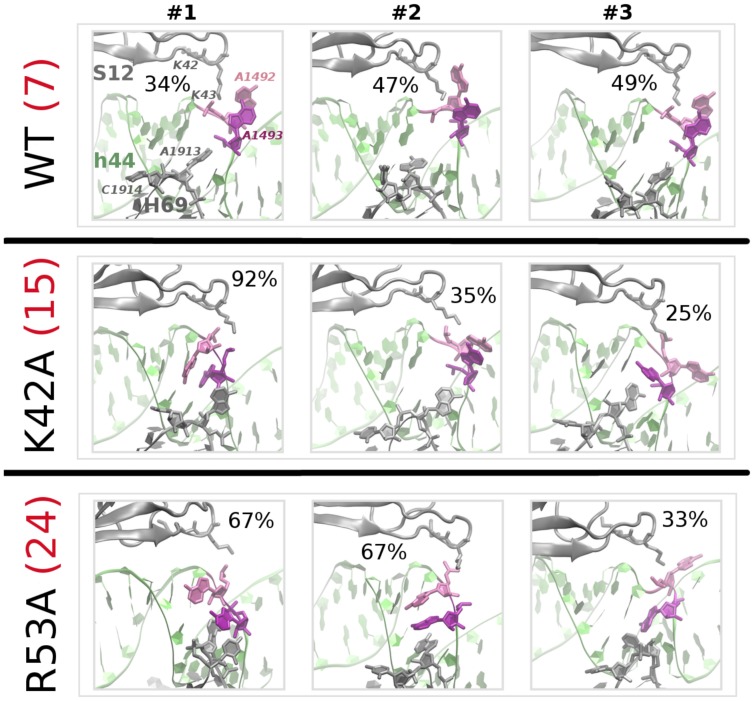
MD conformations of A1492 and A1493 in the uncomplexed A-site. Representatives of final clusters (with occupancies in %) are shown for each simulation run (#1, #2 and #3), in the variants without paromomycin. The h44 helix is shown in green, S12 (above h44) and H69 (below) are grey, A1492 is pink, and A1493 is purple. K42, K43 (S12), A1492 and A1493 (h44), and A1913 and C1914 (H69) are rendered as sticks (hydrogens are not shown). The number of computed structural clusters across all frames (aggregated over all three trajectories of each simulation variant) is shown in red, after the variant label.

**Figure 3:**
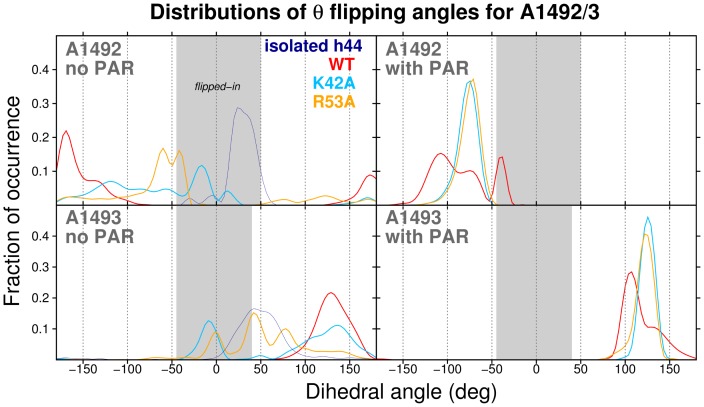
Flipping of A1492 and A1493. Distributions of the pseudo-dihedral angle (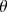) can quantify nucleotide flipping. The shaded region corresponds to *flipped-in* configurations. The 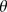 angle and the ‘flipped-in’ 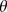 ranges are defined in Fig S1 in File S1) and in the ‘Methods’ section. The data are cumulative from three independent MD trajectories per simulation system variant.

The two key adenines involved in the mRNA decoding process, A1492 and A1493, were found to be highly mobile in all our simulations, with A1492 exhibiting greater mobility (Section S4 in File S1). Moreover, the conformational dynamics of A1492 and A1493 differed in wild-type versus mutant S12 systems. These adenosines exhibited less structural variability in the WT than in the mutants, with a total of seven conformational clusters in WT but at least 15 (and on average 19) distinct clusters in the mutant trajectories (representative conformations are in Fig 2). Furthermore, A1492 and A1493 occupied extra-helical configurations (as in the initial structure) for the entire duration of the WT trajectories, unlike the mutants. Histograms of angular distributions for A1492/1493 (Fig 3) show that both mutants featured a certain population of at least partly *flipped-in* states. The non-negligible occurrence of partly flipped-in configurations of these nucleotides in the mutant trajectories (Fig 3) suggests that complexation with the tRNA anticodon may be less probable for these mutants, versus for the fully flipped-out adenines in the WT system. This trajectory-derived hypothesis is consistent with the higher accuracy of decoding that has been found experimentally for the K42A and R53A mutants [17].

The above result notwithstanding, we emphasize that our sub-microsecond simulation timescale is too short to fully characterize the flipping dynamics of these adenines. We detect only local rearrangements on a nanosecond timescale, and not the multiple global ribosomal motions on microsecond timescales. Indeed, the adenine mobility may depend on the trajectory, as confirmed by the time-evolution of the base flipping angles shown in Fig S1 in File S1. In the mutant S12 systems, we discovered either flipping through the minor groove (which would be expected to occur during the decoding process on the ribosome) or through the major groove (see the representative snapshots in Fig 2, and flipping angles plotted in Fig S1 in File S1).

The true dynamical timescale for adenine motion in the A-site remains unknown. Using replica exchange MD (REMD) simulations, Sanbonmatsu et al. [6] estimated relatively low (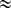
0.8 kcal/mol) energy barriers for flipping of adenines in a ribosomal A-site model, consistent with fast flipping (that work predicted experimental values in the 0.5–5 kcal/mol range). Similarly, fluorescence anisotropy measurements performed on oligonucleotide A-site models suggest that the adenines stack
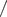
destack on the nanosecond timescale, with rotational correlation times of 0.54
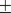
0.15 ns for A1492 [72] and 0.47
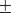
0.09 ns for A1493 [73]. However, NMR carbon spin relaxation and relaxation dispersion studies [74] suggested micro- to millisecond timescales for *full-range* flips of the A-site nucleotides, with the nanosecond timescale corresponding to just *partial* destacking of the adenines. Most recently, Zeng et al. [42], using 2D umbrella sampling simulations, estimated the free energy barrier for full-range flips of both A1492 and A1493 to be relatively high (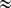
7
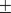
0.3 kcal/mol). Our MD results are most consistent with slow-timescale models for full flipping.

**Figure 4:**
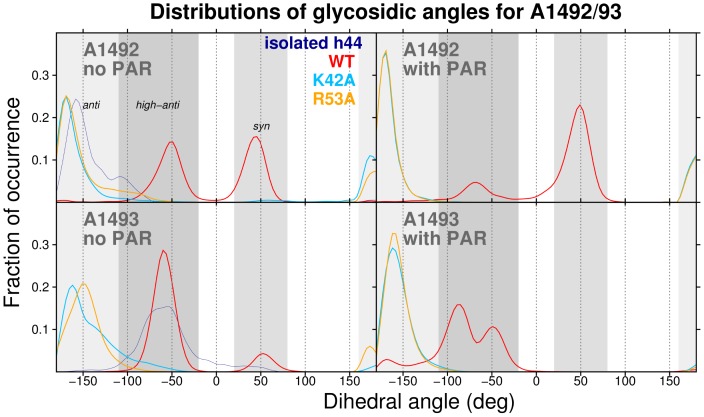
MD-derived distributions of glycosidic angles (in deg) for A1492 and A1493. The *anti*, *high-anti* and *syn* conformational regions are shaded. The data are cumulative from three independent MD trajectories per simulation system variant.

**Figure 5:**
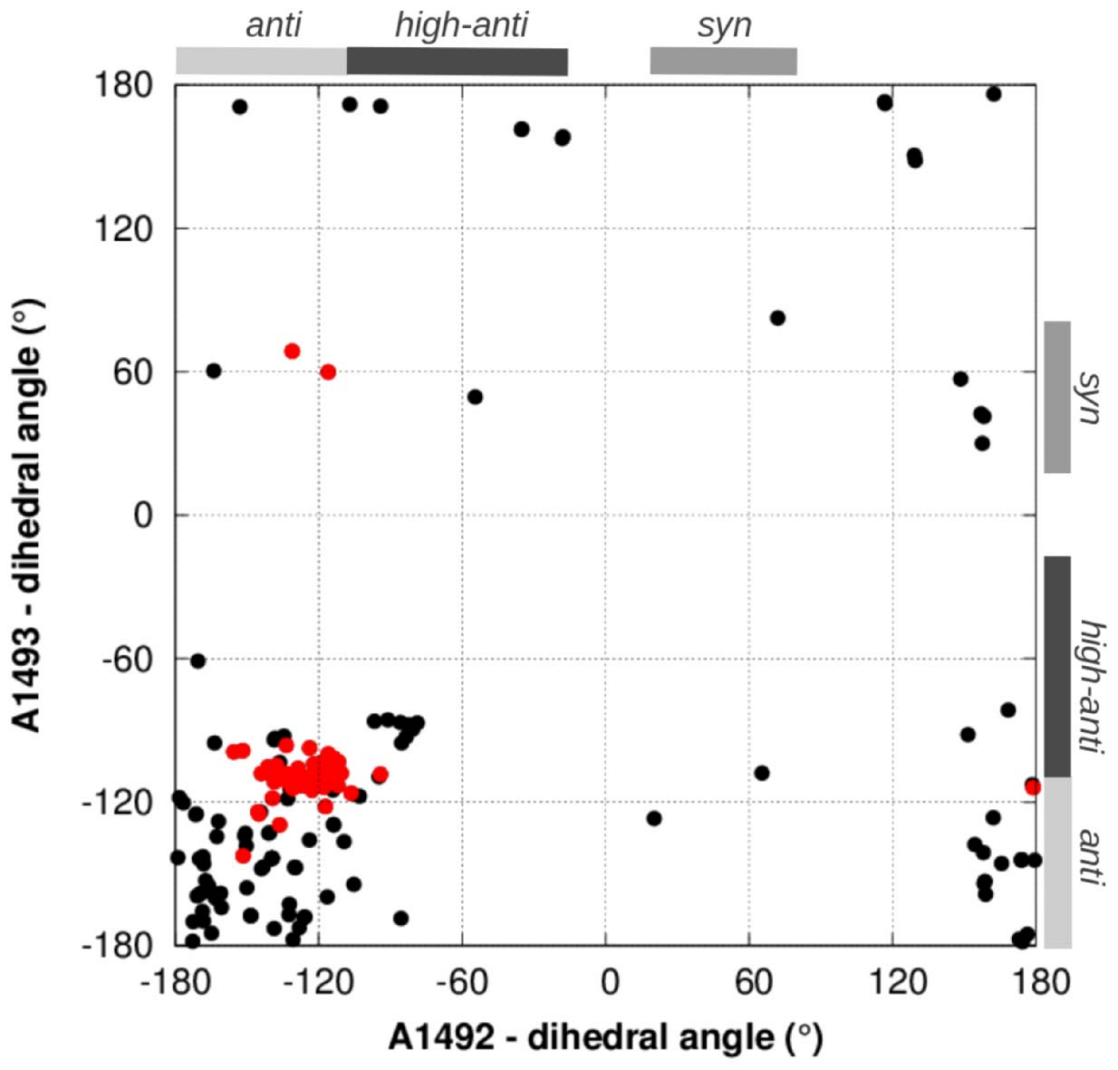
Glycosidic angles of A1492 versus A1493, derived from crystal structures of bacterial ribosomes, with and without A-site tRNA (49 and 126 structures, respectively). Data points for structures with bound A-site tRNA are colored red. The *high-anti*, *anti* and *syn* ranges are indicated.

Interestingly, the distributions of glycosidic angle values (Fig 4) and sugar puckers (Fig S4 in File S1) of A1492
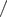
93 differentiate the S12 mutant and wild-type ribosomes. Glycosidic angles (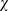) for A-form RNA typically lie in the *anti* range (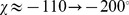), and sugar puckers generally adopt the C3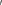 -*endo* conformation. These stereochemical preferences are apparent for the adenines in crystal structures of the bacterial ribosome (Figs 5 and S5 in File S1). However, in all our WT simulations A1492 rotated from an initial *anti* conformation to *syn* or *high-anti* (Fig 4), and this nucleotide more frequently adopted a C2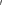 -*endo* sugar pucker, which is atypical for A-form RNA (Fig S4 in File S1). Notably, *syn* and C2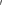 -*endo* conformations of A1492 were also found by Reblova et al. in simulations of the whole helix h44 rRNA, including the A-site [39]. Though not commonplace in structural databases, the *syn* adenine conformations found in our WT (and WT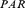) trajectories do exist in some ribosome crystal structures (see Fig 5 and refs [3], [75] and [76]). Surprisingly, and in contrast to our WT simulations, the A1492 *syn* transitions did not occur in any of the mutant trajectories, where both A1492 and A1493 remained exclusively in an *anti* conformation and adopted mostly C3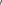 -*endo* sugar puckers (angle distributions are in Fig 4 and Fig S4 in File S1). As described in detail below, these glycosidic angle conformational changes can be attributed to molecular interactions between A1492/93 and the surrounding ribosome.

### Subtle differences in wild-type and mutant A-site dynamics, if paromomycin is bound

**Figure 6:**
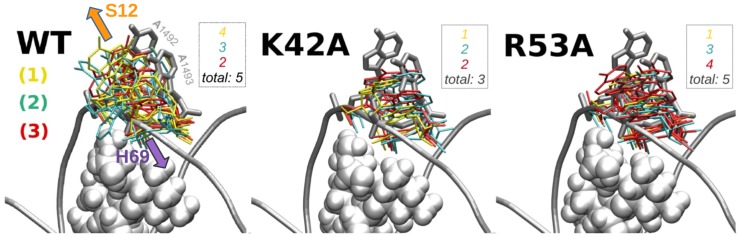
Representative MD conformations of A1492 and A1493 in the variants with paromomycin. Cluster representatives from three MD trajectories (different colors) were superimposed on the crystal structure of the *E. coli* ribosome bound to mRNA and A-site tRNA [7] (PDB 3I1Z). Paromomycin is represented as vdW spheres. The numbers of conformational clusters of A1492 and A1493 for each MD run are listed in each panel as an inset (color-coded as simulation numbers in panel [a]); the total number of conformational clusters is given, from clustering of frames from all three independent MD trajectories for a given simulation variant. Violet and orange arrows indicate the general directions taken by S12 and H69, with respect to A1492 and A1493.

We find that the antibiotic PAR affects the conformational dynamics of the A-site A1492
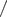
93 in a subtle manner. Our simulations confirm the stability of the antibiotic binding mode in S12-mutated systems, as suggested in experimental work by Sharma et al. [17] (detailed in Section S5 and Fig S6 in File S1). Therefore, we suggest that the mechanism by which the S12 mutations K42A and R53A reduce the miscoding effects of PAR likely does not involve significant changes in the antibiotic orientation in the A-site bulge. In our simulations, the stably-bound PAR molecule did alter certain aspects of the dynamical properties of A1492
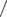
93 in a similar manner for the mutated and wild-type systems (Section S6 in File S1). Beyond this, we also found that the aminoglycoside *differentially* affects A1492/93 dynamics, depending on the presence or absence of the S12 mutations. The difference in adenine conformations in the mutated and WT systems is clearly visible in Fig 6, which shows cluster representatives of these nucleotides in our various simulation systems.

The glycosidic angle distributions in the WT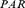 system differ strikingly from the mutant PAR-bound variant systems (Fig 4): In the wild-type trajectory, A1492 is mostly *syn* and A1493 is *high-anti*, versus *anti* 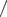 *low-anti* for these adenines in all the mutant trajectories. In ribosomal crystal structures with mRNA and tRNA bound in the A-site, both adenines typically adopt *anti* (nearly *high-anti*) conformations (see experimental data in Fig 5). In contrast, *without* A-site–bound tRNA, the main conformational cluster is located in the *anti* 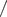 *low-anti* region for both A1492 and A1493. Thus, we propose that *anti* 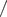 *high-anti* adenine conformations in this region of the ribosome may facilitate the assembly of an mRNA•tRNA•A-site complex. Given this hypothetical model, it would be more difficult for S12 mutants (which sampled mostly *anti* 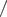 *low-anti* adenine states in our MD), versus the wild-type (which was more often *high-anti*), to form cognate binding interactions with mRNA and tRNA. Fig 6 illustrates the conformational differences between the adenines in the simulated mutants and when A1492/93 are complexed with tRNA anticodon. These results elucidate why translational accuracy is weakly affected by bound PAR in the K42A and R53A mutants.

### The ribosomal surroundings alter A1492 and A1493 dynamics

Several computational and experimental studies have examined A1492 and A1493 mobility in the context of isolated A-site models [6], [9], [22], [38], [40], [41], [77], supplying valuable information about the dynamical properties of A1492 and A1493. Yet, in reality, the adenines are located in a sterically congested ribosomal surrounding (in the long h44 helix), where they interact with protein S12 and with regions of rRNA, such as the H69 hairpin (Fig 1a). Our simulations reveal that the h44 fragment fluctuates much more if this rRNA helix is simulated alone (average RMSF = 1.45 Å) versus in its native ribosomal context (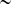
1.0–1.2 Å). These differences were more pronounced for A1492 and A1493 – the mean fluctuations in the isolated h44 were 3.8 Å for A1492 and 3.5 Å for A1493, while in the ribosomal complex these values were 2.1 and 1.6 Å, respectively (Table 1). Our results agree with the intuitive expectation that interactions with the ribosomal surroundings attenuate the dynamics of A1492 and A1493 (and, indeed, the entire h44 helix).

**Table 1:** Fluctuation magnitudes for specific nucleotides in the h44 and H69 helices, as average RMSFs 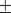 standard deviations in parentheses (Å).

	h44						H69			
	A1408		A1492		A1493		A1913		C1914	
**WT**	0.9	*(0.3)*	2.1	*(0.3)*	1.6	*(0.2)*	1.5	*(0.2)*	2.2	*(0.9)*
**K42A**	0.8	*(0.2)*	2.7	*(0.9)*	2.2	*(0.7)*	1.3	*(0.3)*	1.8	*(0.7)*
**R53A**	0.6	*(0.0)*	3.1	*(0.5)*	2.4	*(0.2)*	1.8	*(0.8)*	1.9	*(0.5)*
**WT** 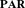	0.6	*(0.1)*	1.8	*(0.2)*	1.4	*(0.3)*	1.4	*(0.6)*	1.8	*(0.8)*
**K42A** 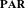	0.8	*(0.2)*	1.2	*(0.1)*	1.0	*(0.1)*	1.5	*(0.5)*	1.4	*(0.2)*
**R53A** 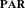	0.7	*(0.1)*	1.4	*(0.2)*	1.2	*(0.2)*	1.6	*(0.5)*	1.5	*(0.1)*
**isolated h44**	1.5	–	3.8	–	3.5	–				

Averages are shown over three simulations per variant; nucleotide numbers are in Fig 1.

Other conformational properties of A1492 and A1493 are also affected by the presence of the ribosomal surrounding. In the isolated h44, A1492 adopts the *anti* glycosidic angle and C3′ -*endo* pucker, while in the WT ribosome systems the C2′ -*endo* and *high-anti*/*syn* are preferentially sampled — compare 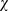 angle and sugar pucker distributions in Fig 4 and Fig S4 in File S1, respectively. The dynamics of A1492/93 base stacking also vary with the simulated model: These adenines stack, on average, 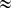
60% of the time in WT systems that include the ribosomal surroundings (Table 2), but only 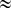
40% of the time in simulations of the isolated h44 helix. This difference likely stems from two factors: (i) the overall greater flexibility of the h44 helix when simulated alone, and (ii) interactions of the A1492/93 pair with protein S12 and H69 rRNA, in the context of the full ribosome, would modify the dynamical behavior of these adenines.

**Table 2:** Stacking frequency for A1492(h44)•A1493(h44) and A1493(h44)•A1913(H69).

	A1492•A1493 (%)	A1493•A1913 (%)
**WT**	57	0
**K42A**	76	15
**R53A**	49	51
**WT** 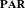 ****	70	15
**K42A** 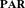	100	55
**R53A** 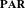	100	45

Values are the percentage of time when the adenines are stacked (data are from three trajectories for each variant). The criterion for stacking is given in the ‘Methods’ section; nucleotide numbers are in Fig 1.

In crystal structures of the bacterial ribosome, A1492 and A1493 are frequently in a *flipped-out* state (see Fig S7 in File S1), which is necessary for ribosome•mRNA•tRNA assembly [5]. However, in most cases this nucleotide conformation is induced by direct interactions with tRNA, antibiotic, and translation factors. Similarly, the *flipped-out* state is more populated in the systems with wild-type S12, even without the bound antibiotic (again, given the caveat mentioned above regarding our limited timescale). In contrast, in bacterial A-site model systems studied via either REMD simulations [6] or NMR experiments (*e.g.*, PDB 1A3M [78]), the *flipped-in* states were thought to be energetically preferred in the absence of antibiotic. In conclusion, we suspect that the tendency for *flipped-out* states of A1492/93 may stem from interactions with the ribosomal surroundings. Possible ways in which the ribosomal surrounding can affect A-site dynamics are discussed in detail below.

### K43 conformational dynamics and A-site mobility

**Figure 7:**
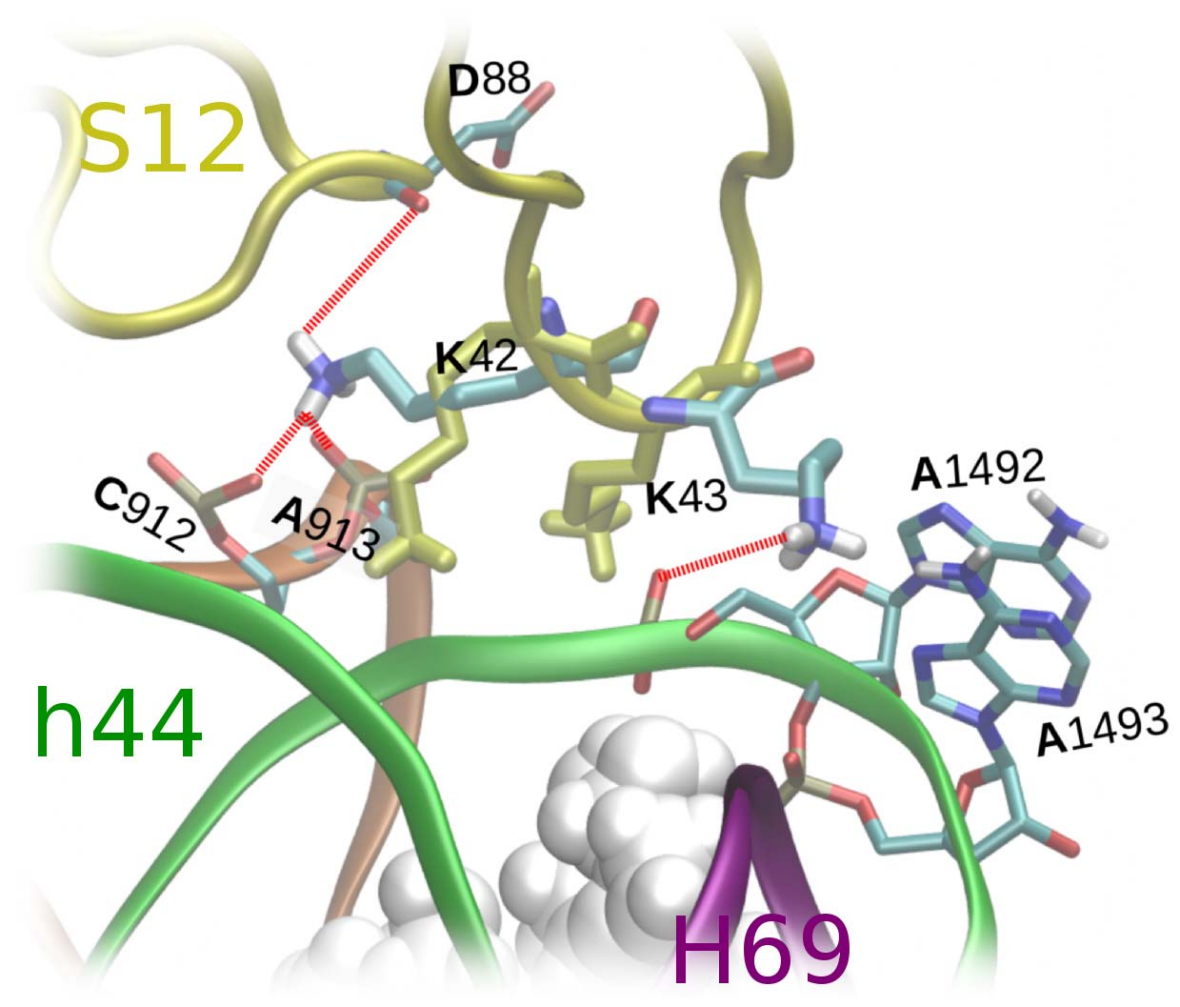
Conformations of K42 and K43. The initial structure is yellow and an exemplary final conformation is colored by atom type. The most frequent H-bonds in the MD trajectories are marked by red dashed lines. Paromomycin is shown as a space-filling vdW representation.

Lysine 43 (K43) of S12 is directly adjacent to A1492 and A1493 (Fig 1a), and is therefore likely to influence the dynamics of these two key nucleotides. Indeed, in our trajectories the K43 side-chain moves distinctly towards A1492, as shown in Fig 7. In crystal structures of the bacterial ribosome, K43 typically occupies a similar position as in our initial MD structure (PDB 2QAL). Interestingly, the alternate conformers of K43 that arose in our trajectories also occur in a few crystallized ribosomes (e.g., PDB 4DH9 [76], 3UXS [79] and 3ZVO [80]), as well as in free 30S subunit structures (e.g., 4JI5 and 4JI7, the latter of which features an S12 point mutation in the PGVR region [11]).

**Figure 8:**
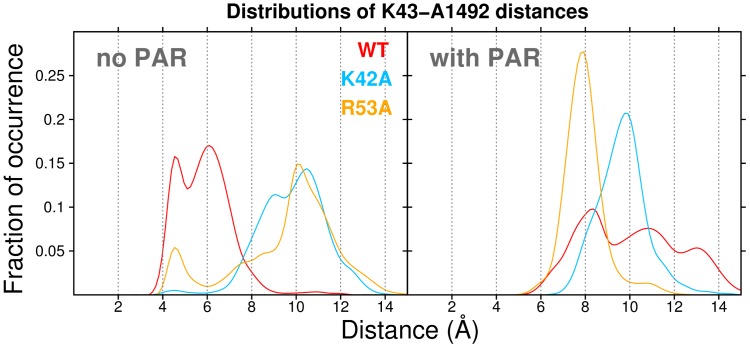
MD-derived distributions of A1492⋅⋅⋅K43 contacts are plotted as distances (in Å) between the centers of mass of the A1492 nucleobase and the K43 side-chain in different simulation variants. The data are aggregated from three independent MD trajectories per simulation variant.

Conformational changes at K43 resulted in interactions of this side-chain with the backbone of helix h44, and in fact mainly with A1492. For 10–70% of the time (depending on the exact trajectory), the primary amine at the end of the flexible K43 side-chain was found to interact with either the phosphate group or, less often, with the ribose oxygens of A1492. The K43 hydrogen bonding partners frequently exchanged atom pairs, presumably due to the significant mobility of K43 and A1492. To compare K43–A1492 interactions in various simulation systems, we calculated (i) distributions of K43⋅⋅⋅⋅A1492 distances and (ii) the average duration of K43⋅⋅⋅⋅h44 H-bonds (as occupancies for each trajectory, averaged over all H-bonding donor⋅⋅⋅acceptor pairs identified in our simulations). The different ribosome simulation systems exhibited different characteristic A1492⋅⋅⋅⋅K43 distances and H-bonds. In the WT systems (without PAR), K43 and A1492 were closer to each other than in the mutants – the WT distance distribution in Fig 8 peaks at 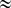
4.5 and 6.0 Å, whereas the mutant peak occurs at 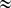
10.0–11.0 Å. As a consequence, K43⋅⋅⋅A1492 H-bonds occurred less frequently in the mutated systems (on average 5.8% for R53A and 7.0% for K42A, versus 14.2% in the WT, Table S2 in File S1).

The structural dynamics of the A1492⋅⋅⋅K43 interaction were also influenced by the antibiotic PAR. The mean distance between these two groups increased in WT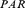 relative to WT (Fig 8). The variance in the WT distance distribution also increased in the presence of PAR, resulting in a broad profile with three weak peaks at 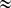 8.5, 11 and 13 Å. Consequently, H-bonds were distinctly weaker in WT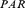 than without PAR (H-bond occupancies of 14.2% versus 5.9%, Table S2 in File S1). In contrast, a single peak dominated the distance distributions for the hyper-accurate mutants K42A and R53A, at 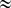
10 and 8 Å respectively. In these cases, relatively large K43⋅⋅⋅⋅A1492 distances were found both with and without PAR; this finding is also reflected in the comparable frequencies of K43⋅⋅⋅ A1492 H-bonds for systems with and without PAR (Table S2 in File S1). These results lead us to suggest that PAR does not significantly influence K43⋅⋅⋅A1492 interactions in our mutant systems, but does have distinct dynamical effects in the wild-type. Thus, we propose that these structural differences may be linked to the experimentally known miscoding effects of PAR, as K43⋅⋅⋅A1492 interactions are diminished in the wild-type system (where PAR causes decoding errors), but are relatively unaffected in our simulations of hyper-accurate S12 mutants (where PAR does not elicit a miscoding effect).

In summary, the K43⋅⋅⋅A1492 interaction properties revaled in our simulations may suffice to differentially affect the dynamical behavior of both A1492 and A1493. Indeed, the K43⋅⋅⋅A1492 interactions described above are likely the reason why the mutant and wild-type systems exhibit different sugar puckers and glycosidic angles for nucleotides A1492 and A1493. For instance, these interactions may be linked to rotations about the glycosidic angle of A1492 in the wild-type systems. Also, K43⋅⋅⋅A1492 contacts may contribute to the recurrence of the *flipped-out* states of A1492 and A1493 in the simulated wild-type variants.

### Coupling of S12 and h44 dynamics through the h27 helix

**Figure 9:**
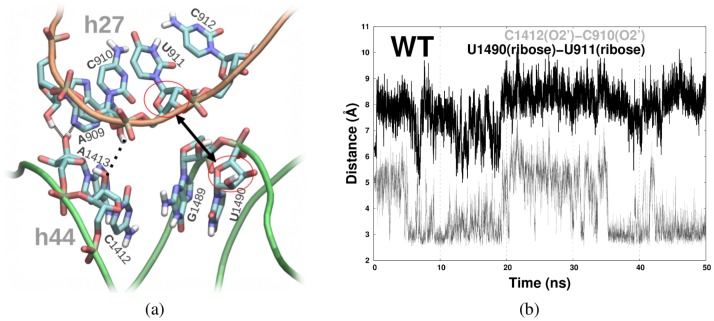
Relative position of h44 and h27. a) The two stable H-bonds (fine dashed line) and one unstable H-bond (dotted line) between h44 and h27 are shown, along with the neighboring regions of h44 and h27 (black arrow). b) The time-evolution of specific h27⋅⋅⋅h44 distances (shown in (a)) are plotted for the wild-type trajectory.

**Figure 10:**
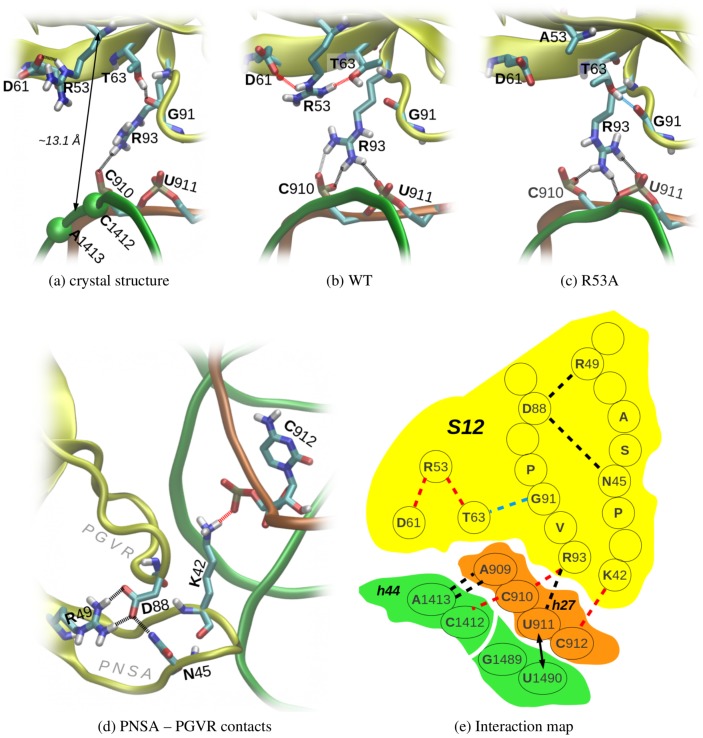
The network of interactions between S12 and rRNA. (a, b, c) Snapshots are shown of the most frequent H-bonds formed between R/A53 or R93 and other regions of the ribosome; only polar hydrogens are shown. Positions of the C 3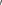 atoms of C1412 and A1413 are indicated. (d) The stable H-bonds between the PNSA and PGVR loops are shown, as is the most frequent H-bond of K42 (which may affect the mutual position of h27 and h44). e) This schematic interaction map for S12 and rRNA shows S12 (yellow), h44 (green), and h27 (orange). The H-bonds observed in MD simulations are denoted by dashed lines as either stable (black), broken/destabilized in some variants (red) or formed upon S12 mutation (light blue). The close neighboring h27 and h44 residues, near the decoding site, are indicated by a black arrow (see text and Fig 9a).

Our simulations reveal that the proximity of the S12 side-chains K42 and K43 to the A-site is one means by which local conformational changes due to the K42A mutation can affect ribosomal A-site dynamics. However, this alone does not explain the structural
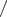
dynamical consequences of mutating S12 at position 53, which lies more distal to the immediate A-site. Instead, our trajectories are consistent with a second coupling pathway, whereby S12 mutations at site 53 detectably influence A-site conformations via a dynamical coupling between S12 and helix h44, with rRNA helix h27 acting as a bridge (orange mark, Fig 1a). Helices h27 and h44 make a few relatively close backbone contacts, some forming stable H-bonds. In particular, the relative configuration of h44 and h27 is stabilized by two H-bonds: A1413(O2′)⋅⋅⋅A909(O2′) and A1413(O2′)⋅⋅⋅A909(N3) (Fig 9a). These interactions stably persisted throughout our MD trajectories, effectively coupling the motion of these two rRNA fragments. Unlike the above interactions, another link between h27 and h44 — namely, a C1412(O2′)⋅⋅⋅C910(O2′) H-bond (Fig 9a) — was only transiently stable, repeatedly forming/breaking in our trajectories (Fig 9b). Most importantly, h27 nucleotides in this latter h27–h44 contact region are also linked to S12, as it creates a salt-bridge with an S12 arginine (R93; Fig 10a). Thus, S12 is indirectly coupled to h44, via the h27 helical region of rRNA.

The two h44 nucleotides C1412 and A1413, involved in this h44⋅⋅⋅h27⋅⋅⋅S12 interaction network, are separated from the A-site bulge by three canonical rRNA base pairs (see nucleotide sequence in Fig 1b). In some of our trajectories, the time evolution of C1412⋅⋅⋅⋅C910(h27) and the other h44–h27 backbone distance, U1490⋅⋅⋅⋅U911, appeared to be correlated (Fig 8b). This implies a possible dynamical signalling pathway between the h44⋅⋅⋅h27⋅⋅⋅S12 contact region and the A-site. Additionally, we discovered that PAR bound in the A-site affects the less stable h27–h44 H-bond mentioned above (C1412(O2′)⋅⋅⋅C910(O2′); Table S3 in File S1): for every ribosomal variant that was simulated, the presence of PAR bound at the A-site was found to distinctly weaken this H-bond. In contrast, we observed the opposite effect — an increase in H-bond stability — in both of the hyper-accurate mutants that we examined (K42A and R53A), compared to the wild-type equivalent. Therefore, we suspect the stability of the h44⋅⋅⋅h27⋅⋅⋅S12 interaction network might be connected to the decoding accuracy.

In summary, we propose that conformational changes may be propagated between the A-site and protein S12, across helix h44, via dynamical coupling of h44⋅⋅⋅h27⋅⋅⋅S12 interactions, and in this way structural changes in the S12 protein indirectly modulate A-site dynamics. Further evidence for a link between S12 dynamics (specifically its R93 side-chain) and instability of the C1412(O2′)⋅⋅⋅C910(O2′) H-bond is elaborated below.

### How do S12 mutations affect the dynamics of the A-site?

While our MD simulations did not reveal major structural changes in S12 near the ribosomal A-site (overall conformational stability of this protein is discussed in Section S2 in File S1), we did find local rearrangements in the amino acid H-bonding network and in the S12⋅⋅⋅rRNA salt-bridges; the latter are known to be important in proper recognition between ribosomal proteins and rRNA [81]. Fig 10e supplies a map of the altered interactions that are presented in this section.

Dynamical rearrangements in S12⋅⋅⋅rRNA interactions are potentially important because they can, for instance, elucidate the structural effects of the R53A mutation, as detected in our simulations. The positive charge of the R53 side-chain appears important for the proper function of this S12 residue: This position features a basic amino acid (Arg or Lys) in 97% of bacterial S12 homologs. However, in the ribosomal conformations used in the present study, position 53 of S12 is quite distant from the A-site and is not directly H-bonded to h44. Thus, we suggest that another physical mechanism (beyond direct electrostatics) also is at play, underlying the effects of the R53A mutation on local dynamics.

The side-chain of R53 drifted from its initial configuration in all of our simulation systems. In the starting crystal structure, the R53 guanidino group directly interacts with only one amino acid – the carboxylate of D61 (Fig 10a). In WT/WT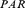 simulations, the R53 side-chain rotated and R53 H-bonded with the hydroxyl oxygen of T63 (Fig 10b is an illustrative snapshot). These H-bonds clearly cannot exist in an R53
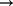
A mutation. However, we found that another H-bond formed in this alanine mutant, between the T63 hydroxyl (which lost its R53 H-bonding partner) and the backbone of G91 from the PGVR motif (Fig 10c). The latter interaction likely contributes to the destabilization of the neighboring, universally conserved R93 (Fig 10c). R93 was found to form salt bridges with the phosphates of C910 and U911 (of helix h27). Intriguingly, R93⋅⋅⋅C910 H-bonds occurred less frequently in the R53A mutant than in wild-type S12; this trend also applies to the K42A mutant regardless of the antibiotic (H-bond occupancies of 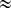
30–35% in the mutants versus 45% in the wild-type). Weakened interactions between R93 and C910 can be associated with rotation of the R93 side-chain, which occurred more frequently in the mutants (compare Fig 10b and c). In summary, this dynamical rearrangement of the positions of R93 (of S12) and R93 interactions with C910 (of h27) may explain the different stabilities of the C1412(O2′)⋅⋅⋅C910(O2′) contact between h44 and h27, as discussed in the previous section.

Less clear is the mechanism by which the K42A mutation could influence R93
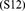
⋅⋅⋅h27 interactions. The coupling may stem from associations between the PNSA and PGVR loops via a stable network of H-bonds, including the D88 carboxylate and the side chains of N45 (PNSA) and R49 (Fig 10d). Conformational changes due to mutation of lysine 42 located near PNSA (Fig 1a and 10e) likely affects the dynamics of PGVR, and thus the conformation of R93. In the case of mutant K42A, electrostatic interactions between the K42 side-chain and the C912 phosphate are important for structural stability of this ribosomal fragment: From bioinformatic database searches, we find that only positively charged amino acids occur at position 42 in all bacterial species for which sequences are available (Lys occurs at this site in 96% of the sequences and Arg in 4%). The S12⋅⋅⋅h27 rRNA interactions (K42⋅⋅⋅C912) that were stable in our MD trajectories without the K42A mutation could not occur in the mutant systems (Fig 10d). This lack of K42(S12)⋅⋅⋅C912(h27) coupling likely modulates the position of h27 and thereby destabilizes interactions between C910 and R93.

We suspect that these altered dynamical properties of R93 (and the S12 protein in general), discovered via our simulations of hyper-accurate mutant systems, would yield different K43⋅⋅⋅A1492 dynamic interaction maps versus the wild-type systems; in this way, the trajectories help elucidate the differences in A1492/93 dynamics in both wild-type and mutant A-site ribosomes.

### Possible functional significance of helix H69 mobility

Despite its key role in ribosome assembly, e.g. as part of the intersubunit bridge [21], little is known about the conformational flexibility of the H69 rRNA helix. The helical stem of H69 was relatively rigid in our simulations, with average RMSDs 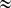
0.8–1.0 (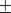
0.2) Å. However, H69 was very mobile in the loop region, particularly at nucleotides A1913 and C1914 (see RMSF values in Table 1), located near the critical A1492 and A1493 nucleotides of h44 (Fig 1abd).

Consistent with our simulations, crystallographic experiments suggest that the loop region of H69 can adopt different conformations (Fig S8 in File S1 and refs [7], [27] and [82]). For instance, in the *E. coli* crystal structure in an intermediate state of ratcheting (PDB 3I1P [7]), both A1913 and C1914 are flipped about the glycosidic bond by nearly 180° with respect to their starting conformations in our simulations (i.e., PDB 2QAM [27]). In both structures, the 50S and 30S subunits were co-crystallized, suggesting that the interactions of the H69 loop with h44 of the 30S subunit do not hinder significant conformational changes of the H69 loop. This is consistent with our MD simulations, where we find high mobility in this rRNA region. Furthermore, in the *D. radiodurans* 50S subunit (PDB 1NKW [82]), crystallized in the absence of 30S, the H69 helix conformation differs significantly from the structures described above (see H69 helices superimposed in Fig S8 in File S1). The *D. radiodurans* 50S subunit features A1913 in a looped-in configuration, which results in heavy-atom RMSDs for H69, between the 50S-only crystal structure (PDB 1NKW) and the full 70S ribosome structure (PDB 2QAM), being as high as 3.1 Å. Also, ‘closed’ configurations of the H69 hairpin, similar to what occurs in the 1NKW crystal, have been found in the full 50S subunit via dimethyl sulfate (DMS) probing experiments at acidic pHs [23]. Taken together, our observations suggest that coupling of H69 (of the 50S subunit) with h44 (of the 30S subunit), which form the B2a inter-subunit bridge, may promote the *flipped-out* state of A1913. Also interesting, among all our simulations the average H69 structure remained closest to its initial conformation in the wild-type trajectories, with a mean RMSD of 1.7 Å (Tab S1 in File S1). This result suggests that antibiotics bound to the A-site, as well as S12 mutations, could also affect ribosomal translation by altering the conformational dynamics of H69. In summary, we suspect that the coupling between the conformational states
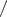
dynamics of A1913 (in helix H69) and A1493 (in helix h44, at the A-site), as detected in our simulations, is a result of the close spatial proximity of these nucleotides at the junction of rRNA structural elements (Fig 1a).

### Influence of helix H69⋅⋅⋅h44 interactions on A-site flexibility

The nucleotides in the hairpin loop of H69 interacted with h44 in most of our simulations. Specifically, stacking interactions between A1913 (of H69) and A1493 (of h44) repeatedly formed and broke, as shown for sample conformations in Fig 2; the stability of these H69⋅⋅⋅h44 interactions depended on the exact simulated variant. In the mutants either with or without PAR, A1493 and A1913 stacked more frequently than in the wild-type systems. With PAR bound, stacking occurred on average 50% of time in the mutant systems versus 15% in WT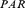 (Table 2). Also, mutated A-sites in the absence of PAR still exhibited A1493•A1913 stacking (15% of the time for K42A and 51% for R53A), unlike the WT without PAR (which was 0%). Thus, S12 mutations appear to have the dynamical effect of enhancing A1493•A1913 stacking interactions.

The above tendency in the S12 mutant systems can be understood in terms of an overall shift of the A1492/93 nucleobases away from S12 and towards H69, with respect to wild-type systems. This shift for the mutants with bound PAR is illustrated via clustering results in Fig. 6; the effect is also manifested in the systems without PAR as larger A1492–K43 distances (Fig 8). Interestingly, in crystal structures of the bacterial small ribosomal subunit Demirci et al. noticed that bound streptomycin causes a lateral shift of the A-site towards S12 protein (and helix 18) [20], and the hyper-accurate mutations P90W and P90L yield basically the opposite effect [11]. A streptomycin-induced shift toward S12 was found to stabilize interactions of near-cognate tRNA codons with mRNA, while destabilizing the cognate tRNA codons, and thereby resulting in miscoding [20]. We did not observe such significant conformational changes of the h44 backbone in our MD simulations; we emphasize that the studies by Demirci et al. were performed on isolated 30S subunits, whereas our simulation systems also contain the (structurally stabilizing) 50S fragments, including the H69 helix. The crystallographically characterized conformational shifts are consistent with our observations from MD trajectories of the *hyper-accurate* mutants (K42A and R53A), where we find that A1492 and A1493 shift further from S12 (and closer to H69).

Also, the higher frequencies of A1493⋅⋅⋅A1913 interactions in the simulated mutants – versus the wild-type – may be linked to the wider range of A1493 flipping in the uncomplexed mutants versus WT (Fig 3). In WT trajectories, A1493 sampled only a narrow range of 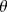 values, corresponding to a distinct peak in the histogram (at 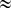
130°). The increased A1913•A1493 stacking in the mutants is likely linked to the occurence of *flipped-in* conformations of A1492 and A1493, and may stabilize the *flipped-in* state of A1493. Therefore, we predict that A1913•A1493 stacking contributes to hyper-accuracy of the K42A and R53A mutants by slowing adenine flipping during the mRNA decoding process. To conclude, A1493•A1913 stacking interactions may favor A1493 states that are less amenable to proper assembly of a complex between mRNA
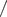
tRNA and the A-site.

Intriguingly, our MD studies reveal that the binding of PAR, which causes miscoding, corresponds to a similar conformational/dynamical effect as S12 mutations, which have the opposite biological consequence (hyper-accuracy). Specifically, binding of PAR in the A-site (similarly as the mutations) increases the propensity for A1913•A1493 stacking in the WT and in the K42A mutant. For instance, A1493 and A1913 stacked on average 15% of the time in WT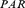, but did not stack at all in the WT trajectory (Table 2). Ribosomal 30S crystal structures of Demirci et al. [20] with PAR bound in the A-site do not show a significant deformation of the h44 backbone (unlike the analogical streptomycin-bound complexes, discussed above). Therefore, interpretation of the adenines' shift toward the H69 helix observed in our PAR-bound MD systems is unclear. So further studies will be required to elucidate the link between A1913⋅⋅⋅A1493 interactions and the miscoding
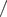
hyper-accuracy phenotype. In the case of A-site–bound PAR, another (stronger) factor likely dominates, e.g. enforcing A1492 and A1493 to *flipped-out* conformations, thereby eliciting the miscoding phenotype. Furthermore, the dynamics of A1913⋅⋅⋅A1493 interactions in the decoding process also will be modulated by large-scale conformational changes across the entire ribosome (i.e., at relatively low resolution) [1], [48], beyond the local dynamics of the A-site region that we describe here at high resolution.

Helix H69 has been shown to be inessential for correct mRNA decoding in the bacterial ribosome [25]: Genetic deletion of H69 – though lethal *in vivo* due to problems with subunit assembly, peptide release, and recycling – still allows for ‘smooth’ translation *in vitro*. Relatively little is known about mechanistic changes in mutated or aminoglycoside-bound ribosomes, or how H69⋅⋅⋅⋅h44 interactions affect translational processivity. Although helix H69 is not crucial for translation, perturbations in its structure – such as introduction of larger nucleobases (m
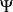
1915A [26]) or nucleotide deletion (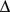
A1916 [83]) – do perturb the decoding process, likely via altered H69–h44 interactions that result from changes in the shape of these rRNA fragments upon mutagenesis. Taken together, these observations agree with our MD findings: in the wild-type system (without PAR) we did not observe A1913⋅⋅⋅A1493 stacking, consistent with interactions between A1493 and A1913 being dispensable to the mRNA decoding process. Aminoglycoside binding to the A-site, and
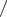
or S12 mutations, may ‘switch on’ these A1913⋅⋅⋅A1493 interactions which, in turn, interfere with the dynamics of the mRNA decoding. Such a model is most consistent with both our MD simulations and the fact that helix H69 can be deleted in experiments without affecting ribosomal translation [25].

## Summary and Conclusions

To explore the flexibility of the ribosomal 30S subunit A-site, we performed 18 atomistic MD simulations of a region of the ribosome that includes part of rRNA helix h44, helix H69, and the protein S12. With an aggregate length of 0.9 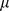
s, these trajectories allowed us to extensively sample the structural and dynamical effects of S12 point mutations (the hyper-accurate K42A and R53A), as well as of a bound aminoglycoside antibiotic (paromomycin). In order to simulate the dynamics of the A-site in atomic detail, for a system as large as the ribosome, we took a ‘restrained fragment’ approach that implicitly accounts for the boundary between the A-site and the surrounding ribosome.

Interactions with the ribosomal surroundings, in particular with K43 (of S12) and A1913 (of H69), were found to significantly restrict the motion of A-site nucleotides A1492 and A1493. Various conformational properties of these nucleotides were also influenced; for example, the glycosidic angles and sugar pucker conformations of the adenines differed in the trajectories of wild-type and mutant S12 systems. Based on our simulations, we suggest that (A-site)⋅⋅⋅S12 and (A-site)⋅⋅⋅H69 interactions — i.e., A-site dynamics in the context of the entire ribosome — may lengthen the timescale for flipping of A-site nucleotides, versus simulation systems comprised of fragment-only models. In our wild-type S12 simulations, K43⋅⋅⋅A1492 contacts were the most probable factor that would preclude flipping-in of the two A-site adenines. In the mutant S12 systems, these interactions were less frequent and A1492/93 exhibited flipping behavior. These differing conformational preferences would be expected to make the assembly of a complex between A1492/93 with an A-site tRNA less probable in the mutant systems than in the wild-type ribosomes, thereby explaining the hyper-accuracy phenotype of the S12 mutant systems that we simulated.

The binding mode of paromomycin in the A-site was not significantly destabilized in any of the S12 mutants that we simulated, in accord with experimental data [17]. However, the antibiotic did differentially influence K43⋅⋅⋅A1492 interactions: In the wild-type ribosome, the frequency of H-bonds between these two residues was noticeably reduced if paromomycin was bound, thus implicating this mechanistic feature as a contributing factor to the miscoding effect of the antibiotic. On the contrary, K43⋅⋅⋅A1492 interactions in our S12 mutant systems were unchanged by paromomycin binding, consistent with the known resistance of hyper-accurate mutations to aminoglycoside-related miscoding.

We also found that two factors — the binding of paromomycin, or the existence of S12 mutations — enhanced the propensity for stacking between A1493 (of h44) and A1913 (of H69). Such stacking interactions did not occur in the wild-type system without paromomycin. This finding can be explained by a conformational shift of adenines 1492/93 away from S12 and toward H69. This model is consistent with crystallographic results, which suggest that such conformational changes of h44 (in a similar direction as we find) may be associated with the hyper-accurate phenotype. Additionally, because experimental data suggest that h44⋅⋅⋅H69 interactions are not crucial for mRNA decoding in wild-type bacteria [25], we suspect that S12 mutations and antibiotic binding could perturb the mRNA decoding process by inducing these interactions (specific mechanisms for such effects remain unknown).

Finally, we determined that the conformational differences of A1492/93 in wild-type versus S12 mutants may arise from rearrangements in the hydrogen bonding patterns of S12 and the neighboring rRNA. These changes to structure and stability of hydrogen bonding (and other noncovalent interactions) can be propagated into larger-scale, allosteric changes because the dynamics of protein S12 and helix h44 are coupled via the h27 helix. Overall, our results illuminate – in atomic detail – the mechanistic basis for the effects of S12 protein mutations and aminoglycoside antibiotic binding on the local conformational dynamics of the A-site of the bacterial ribosome.

## Supporting Information

File S1
**Supporting files.** **Figure S1**, Flipping of A1492 and A1493 (without the antibiotic) during MD simulations. a) The 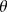 pseudo-dihedral angle used to describe flipping (here shown for A1492) defined by the four pseudoatoms: CM1 – centre of mass of the neighboring base pair (C1409 and G1491), CM2 – centre of mass of the ribose of the neighboring nucleotide (G1491), CM3 – centre of mass of the flipping nucleotide ribose (A1492), CM4 – centre of mass of the flipping nucleobase (A1492). The values of 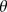 for A1492 and A1493 versus time in the variants: b) WT, c) K42A and d) R53A in each MD trajectory (the simulation numbers are shown in parentheses in [b]). in the variants: b) WT, c) K42A and d) R53A in each MD trajectory (the simulation numbers are shown in parentheses in [b]). The typical direction of A1492 and A1493 flipping movement during mRNA decoding process (through the minor groove of the rRNA helix) is indicated by arrows in panel (b). **Figure S2**, Convergence of MD simulations for the wild-type systems with and without paromomycin. a) Evolution of RMSD from the starting structures for the solute heavy atoms in each of the 3 simulations in WT and WT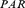 variants. b)-d) Cumulative RMS fluctuations. The average fluctuations were calculated for the selected subsets of residues as a function of time: b) the h44 helix, c) ‘head’ of the S12 protein and d) the H69 helix. Panel (e) shows the membership of mutual A1492 and A1493 conformations in time to different clusters in three WT simulations (without paromomycin). Different colors mark different clusters. Clustering was done for conformations gathered from all 3 trajectories, with 3.5 Å radius (see ‘Methods’ in the main text). **Figure S3**, Structural stability in the simulated systems. a) RMSD (P/C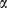) versus time for h44 and H69 helices and the “tail” of the S12 protein in the chosen MD simulations. The structures with the highest RMSD in the trajectories selected from those presented in (a): b) h44 and c) S12 and the three nucleotides mentioned in the text: U911 – in light green, C912 – blue, A913 – red; red arrow indicates the direction of conformational change from the initial structure. **Figure S4**, Distributions of sugar pucker phase for A1492 and A1493 in MD simulations. The regions corresponding to C2′- *endo* and C3′- *endo* configurations are shaded. The data are cumulative from three independent MD trajectories per simulation variant. **Figure S5**, Sugar pucker phase of A1492 vs A1493 gathered from 170 crystal structures of the bacterial ribosome. The C2′- *endo* and C3′- *endo* ranges are indicated. **Figure S6**, Structural stability of paromomycin in MD simulations: a) RMSD in time for the ring IV of paromomycin (see Fig 1e, main text) in the two simulations: one of WT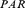 with stable PAR and the other of R53A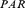 variant, with exceptionally unstable antibiotic. b) Conformational change of paromomycin in the R53A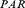 trajectory: the initial structure – in yellow, the conformation with the highest RMSD – colored by atom type. **Figure S7**, Positions of A1492 vs A1493 in crystal structures of the bacterial ribosomes. The plots of C1′(C1409)-C1′(G1491)-C1′(A1492)-N9(A1492) versus C1′(C1407)-C1′(G1494)-C1′(A1493)-N9(A1493) dihedral angles. These angles were shown to characterize well flipping of the nucleotides in bulges [84] and they are analogical to the 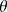 angles defined in Fig S1a in File S1. The values close to 0 correspond with the flipped-in conformations, while the values close to 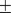
180 – the flipped-out states. The arrows indicate the two directions of flipping-out: through the minor and major groove (marked by continuous and dotted lines, respectively). Data are gathered from 167 structures. **Figure S8**, Comparison of H69 conformations in different crystal structures of bacterial ribosomes. Abbreviations: E. c. – *E. coli*, D. r. – *D. radiodurans*. PDB codes are given. **Sections S1**–**S6**, Describe the force field considerations (Section S1), dynamics and stability of the S12 protein (Section S2), overall mobility of the h44 and H69 helices (Section S3), fluctuations of A1492 and A1493 (Section S4), conformations of paromomycin (Section S5), and how the antibiotic bound in the A-site affects the conformational dynamics of A1492 and A1493 (Section S6). **Table S1**, Deviations from the initial configuration during MD for the chosen subsystems: averages of RMSD (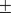 standard deviation, in Å), calculated for P and C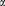 atoms with respect to the initial structure. For each simulation variant the data from 3 MD trajectories are averaged over time. **Table S2**, Duration of H-bonds created between N3 nitrogen of K43 and h44 nucleotides (expressed as % of trajectory time). For each MD simulation run it is calculated as an average of occupancies for H-bonds of K43(N3) with 13 hydrogen acceptors: G1491 (O1P, O3′), A1492 (O1P, O2P, O2′, O4′, O5′, N3, N7), A1493 (O1P, O2P, N1, N7). The data are also averaged over 3 trajectories for each variant (standard deviations in parentheses). One WT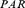 simulation is excluded from averaging (as in this case the K43⋅⋅⋅⋅h44 interactions are not formed) and it is treated as an exception. **Table S3**, Percentage of time when H-bond between C1412(O2′) and C910(O2′) atoms is formed during MD simulations (data are from three trajectories for each variant).(PDF)
