# Supplementary material for: Interplay of the Bacterial Ribosomal A-Site, S12 Protein Mutations and Paromomycin Binding: A Molecular Dynamics Study
Source: PLoS One. 2014 Nov 7;9(11):e111811. doi: 10.1371/journal.pone.0111811 (PMC4224418; doi:10.1371/journal.pone.0111811)
Supplement: File S1 — Supporting files. Figure S1, Flipping of A1492 and A1493 (without the antibiotic) during MD simulations. a) The pseudo-dihedral angle used to describe flipping (here shown for A1492) defined by the four pseudoatoms: CM1 – centre of mass of the neighboring base pair (C1409 and G1491), CM2 – centre of mass of the ribose of the neighboring nucleotide (G1491), CM3 – centre of mass of the flipping nucleotide ribose (A1492), CM4 – centre of mass of the flipping nucleobase (A1492). The values of for A1492 and A1493 versus time in the variants: b) WT, c) K42A and d) R53A in each MD trajectory (the simulation numbers are shown in parentheses in [b]). in the variants: b) WT, c) K42A and d) R53A in each MD trajectory (the simulation numbers are shown in parentheses in [b]). The typical direction of A1492 and A1493 flipping movement during mRNA decoding process (through the minor groove of the rRNA helix) is indicated by arrows in panel (b). Figure S2, Convergence of MD simulations for the wild-type systems with and without paromomycin. a) Evolution of RMSD from the starting structures for the solute heavy atoms in each of the 3 simulations in WT and WT variants. b)-d) Cumulative RMS fluctuations. The average fluctuations were calculated for the selected subsets of residues as a function of time: b) the h44 helix, c) ‘head’ of the S12 protein and d) the H69 helix. Panel (e) shows the membership of mutual A1492 and A1493 conformations in time to different clusters in three WT simulations (without paromomycin). Different colors mark different clusters. Clustering was done for conformations gathered from all 3 trajectories, with 3.5 Å radius (see ‘Methods’ in the main text). Figure S3, Structural stability in the simulated systems. a) RMSD (P/C) versus time for h44 and H69 helices and the “tail” of the S12 protein in the chosen MD simulations. The structures with the highest RMSD in the trajectories selected from those presented in (a): b) h44 and c) S12 and the three nucleotides [file pone.0111811.s001.pdf]

Supporting Information for
Interplay of the bacterial ribosomal A-site, S12 protein mutations and paromomycin binding: A molecular dynamics study

Joanna Panecka[1,2], Cameron Mura[3], Joanna Trylska[4]

[1]Division of Biophysics, Institute of Experimental Physics, University of Warsaw, Żwirki i Wigury 93, 02-089 Warsaw, Poland; [2]Interdisciplinary Centre for Mathematical and Computational Modelling, University of Warsaw, Pawińskiego 5a, 02-106 Warsaw, Poland; [3]Department of Chemistry, University of Virginia, Charlottesville, VA 22904-4319, USA; [4]Centre of New Technologies, University of Warsaw, Banacha 2c, 02-097 Warsaw, Poland

S1 Force field considerations

The parmbsc0 parameter set (1) was not used in our main set of simulations. This force-field modification was originally intended to prevent 'irreversible' α/γ flips in the DNA backbone. In an ideal A-form RNA helix, α and γ values oscillate about -70° and 55°, respectively (2). Whether or not parmbsc0 is the best choice for simulating RNA remains unclear, especially if one seeks to study nucleotide movements in various RNA motifs, such as the A-site bulge. Others have reported similar performances of the parm99 and parmbsc0 force-fields for RNA systems (3, 4), showing that α/γ flips are reversible if a simulation lasts sufficiently long. Also, reversible α/γ changes in the h44 backbone (C1411) were reported in previous MD studies utilizing parm99 (5). Finally, overall backbone RMSD values indicate that the parm99-simulated S12 systems are structurally stable (Tab S1). To compare the two force-fields, we performed short (20 ns) MD simulations of the h44 fragment excised from the full system. Although we observed that α/γ flips occurred to a lesser extent with parmbsc0 than with parm99, we note that in the latter case we found the flips to be generally reversible. Also, flipping of the adenine bases was similar in both force-field variants, though in parm99 we detect greater conformational variability (data not shown).

Another modification of the Amber force-field (6), to improve the treatment of glycosidic angles, has not been used here because our systems include modified nucleobases which have not yet been parameterized. To assess any tendency of our simulations to yield artefactual 'ladder' structures (6), we fitted a linear function to the average χ angles for h44 and H69 in all systems (averaging only over all nucleotides paired with standard Watson-Crick geometry). We found, during 50 ns of MD, the maximal increase of χ values to be 10° (for h44 in the R53A$_{PAR}$

system) and 14° for H69 (in WT_{PAR}). The mean χ values remained in the *anti* region during the whole trajectory, thus eliminating the formation of RNA 'ladders' as a potential concern.

We also have to note that in our MD simulations we observed a slight tendency towards *high-anti* conformations, known for the parm99 (7) force-field, for the bulged-out nucleotides A1492 and A1493. However, Fig 4 (main text) shows that the *high-anti* state occurred only in the non-mutated variants, and less frequently in the isolated h44 helix. Thus, the differences we observed in the glycosidic angle distributions likely can be ascribed to *bona fide* changes in the molecular dynamics of our systems, rather than to force-field artefacts.

S2 Dynamics and stability of the S12 protein

We inspected the dynamical behavior of the S12 protein. The "head" of S12 (see Fig 1ac, main text), which is located close to the PAR binding site, was structurally stable, with average RMSDs of 1.0–1.1 Å and standard deviations about 0.1–0.2 Å (Tab S1). The structure of the β-sheet core of S12 was well preserved during the simulations, with the total occupancy (value averaged over all residues of the S12 'head') of β-sheet in a range 40–42% in all of the systems. Most of the S12 conformational changes occurred in the N-terminal "tail" region (Fig S3c). RMSDs for heavy atoms of S12 "tail" with respect to the crystal structure are higher than for the other fragments of the systems, between 1.7 and 2.2 Å, similarly as their standard deviations that fall in a range of 0.3–0.4 Å (Tab S1).

In the majority of the simulations the N-terminal tail of S12 moved systematically. Especially, in one of the WT_{PAR} simulation the conformation of the S12 tail changed distinctly. The configuration from WT_{PAR}, with the highest RMSD with respect to the initial structure, was shown in Fig S3c. Interestingly, in many of the simulations (in particular – all native variants) the movement of the tail occurred in similar direction, towards the residues U911–A913, which are shown in Fig S3c. However, we have to note that the long extension of S12 is natively unstructured (such fragments occur also in the other ribosomal proteins (8)), and this justifies its significant mobility. Also, the S12 tail is located rather far from the region that we focus on, namely the interface between h44, H69 and S12.

S3 Overall mobility of the h44 and H69 helices

The RMSDs in Tab S1 show that H69 was the most mobile rRNA fragment in all the simulated systems. In particular, H69 changed its position with respect to the h44 helix. The average RMSDs for H69 are in the range of 1.7–2.1 Å and their standard deviations reach even 0.5 Å. This proves high mobility of H69, precisely – of its looped fragment located close to the A-site.

Also, in some cases the backbone of helix 44 relaxed to the conformation differing visibly from that in the initial structure. For instance, the conformation from one of the R53A simulations with the highest RMSD with respect to the crystal structure is shown in Fig S3b. Tab S1 shows that the average RMSDs for h44 are higher without PAR (e.g., RMSD_{ave} equals 1.3 Å for WT_{PAR} and 1.6 Å for WT). It probably results from the limited mobility of the whole helix 44 due to the antibiotic binding. And further, this allows for the formation of more stable contacts between the h44 and H69 nucleotides in the presence of PAR, which is discussed in the main text.

S4 A1492 fluctuates more than A1493

A1492 fluctuated more than A1493 in all of the simulated systems (see RMSF values in Tab 1, main text). In particular, multiple discrete flipped-out states of A1492 occurred, whereas the conformational variability of A1493 was not that great in terms of θ pseudo-dihedrals (Fig 3, main text). These results are consistent with the crystallographic work of Shandrick et al. (9), who found two equally probable (and thus iso-energetic) conformations of A1492 in the absence of PAR – *inside* and *outside* the A-site bulge. The high mobility of A1492 in our simulations suggests that, indeed, this nucleotide likely can exchange between two distinct experimentally observed configurations. Yet, in our MD simulations it seems that the free energy landscape for A1492 flipping was rather flat, as we observed a broad range of *flipped-in* and *flipped-out* states in θ distributions in Fig 3 of the main text. Thus, the two distinct conformations in the crystal structure (determined at a temperature of 100 K) may correspond to trapped states from this distribution.

Also, the higher mobility of A1492 in the context of the ribosome (this work) differed from previous REMD simulations of an A-site model (10), where the flipping of A1493 in the isolated A-site was roughly 5× more frequent than of A1492. That work estimated the energetic barrier for A1492 to be higher than that of A1493 (1.68 vs 1.38 kcal/mol). Taken together, the REMD simulations (10) and the experiments (9) suggest that A1492 has a relatively high kinetic barrier to flipping between two equiprobable conformers. The discrepancy between our results and earlier findings may largely stem from the fact that the REMD simulations and experiments were performed on small, isolated models of the A-site, whereas our present calculations include the local ribosomal environment. As discussed in the main text, we found that the ribosomal surrounding exerts a non-negligible influence on the dynamics of A-site moieties. When we examined the flipping of adenines in MD of just the isolated h44 helix (distributions in Fig 4, main text), the range of flipping is indeed broader for A1493 than for A1492, in compliance with these earlier studies by others (9, 10).

The relative magnitudes of A1492/1493 fluctuations in our simulations (see Tab 1, main text) roughly agree with the temperature factors from the crystal structure that was taken as our starting point. Specifically, the B-factor values (PDB 2QAL (11)) are 153 for the nucleobase of A1492 versus 137 Å^2 for A1493. However, there are caveats on the interpretation of these values, as the B-factors vary from one crystal structure to another and differ between the ribosomal subunits in the same diffraction experiment (PDB 2QAN and 2QAL (11)), due to the conformational heterogeneity across the lattice and between the two 30S subunits found in the same asymmetric unit of these crystals.

S5 Conformations of paromomycin

We did not observe any specific influence of the mutations on the conformation of paromomycin in the A-site. The antibiotic was exceptionally stable in most of the simulations, with RMSF (for heavy atoms) of about 0.3 Å. Only in one R53A$_{PAR}$ simulation we did note a slight conformational change of paromomycin (Fig S6). Ring IV was quite mobile and one of the amine groups (N46, shown in Fig 1e, main text) rotated interacting either with phosphate of U1490 or G1489. Next, it lost these interactions and its solvent accessible surface area increased from

about 230 to 270 Å^2. The mobility of this sugar ring of PAR was also observed in the other MD simulations (12, 13). Additionally, the position of the pair of uracils 1406 and 1495 (see Fig 1b, main text), located in the aminoglycoside-binding cleft was stable in almost all of the simulations (the only exception is one K42A$_{PAR}$ simulation). It is consistent with the observed stability of PAR, as U1406 and U1495 are important for the stable binding the aminoglycoside antibiotics in the A- site (12, 13, 14).

S6 The antibiotic bound in the A-site affects the conformational dynamics of A1492 and A1493 in various ways

The RMSFs for A1492 and A1493 in (Tab 1, main text) show that in each variant (either mutated or native) PAR restricted the movement of these adenines. Also, the clustering analysis confirmed that with PAR bound the configurations of A1492 and A1493 were less variable than in the absence of the aminoglycoside. This is reflected by the numbers of clusters (Fig 2 and 6, main text): when the antibiotic was bound we obtained 3–5 clusters, and 7–24 in the opposite case. Also, fluorescence studies reported a decreased mobility of the adenines as a result of PAR binding to the A-site model (15). The lower mobility is expected because, as proven by the crystal and experimental structures (16, 17, 18, 19), and theoretical data (12, 14), the bound aminoglycoside occupies the space inside the major groove that would normally be accessible for the adenines, and the latter adopt extra-helical configurations. Indeed, in our simulations the adenines were located rather outside of the A-site bulge, as shown in Fig 6 (main text) and confirmed by the distribution of θ pseudo-dihedral angles in Fig 3, (main text). Furthermore, binding of PAR also stabilized stacking between the adenines (Tab 2, main text). For example, in the mutants the adenines were stacked 100% of time when the antibiotic was bound. Also, in all the variants the presence of aminoglycoside increased the occurrence of C3'-*endo* sugar pucker, characteristic of regular A-type helices (Fig S4).

Finally, in the wild-type variant we noted different conformational tendencies for A1493 depending on the presence of PAR. The glycosidic angle distributions for A1493 presented in Fig 4 in the main text show additional peak at about $\approx -90°$ (*high-anti*) in WT$_{PAR}$ instead of just one maximum at $\approx -60°$ in the absence of PAR. Surprisingly, the peak found only in the WT$_{PAR}$ systems is close to the value frequently acquired in ribosomes bound to mRNA and A-site tRNA (see experimental data in Fig 5 in the main text). Thus, it is possible that PAR also increases the acceptance of non-cognate anticodons by favoring this *high-anti* conformation of A1493. This agrees with the observation that in contrast to WT$_{PAR}$, the *high-anti* conformation of A1493 did not occur in the simulations of the mutated, hyper-accurate, systems (Fig 4 in the main text).

References

1. Perez A, Marchan I, Svozil D, Sponer J, Cheatham TE, et al. (2007) Refinement of the AMBER force field for nucleic acids: improving the description of alpha/gamma conformers. Biophys J 92: 3817–3829.

2. Blackburn CM, Gait MJ, editors (1996) Nucleic acids in chemistry and biology. Oxford University Press.

3. Besseova I, Otyepka M, Reblova K, Sponer J (2009) Dependence of A-RNA simulations on the choice of the force field and salt strength. Phys Chem Chem Phys 11: 10701–10711.

4. Reblova K, Strelcova Z, Kulhanek P, Besseova I, Mathews DH, et al. (2010) An RNA molecular switch: Intrinsic flexibility of 23S rRNA helices 40 and 68 5'-UAA/5'-GAN internal loops studied by molecular dynamics methods. J Chem Theory Comput 2010: 910–929.

5. Reblova K, Lankas F, Razga F, Krasovska MV, Koca J, et al. (2006) Structure, dynamics, and elasticity of free 16s rRNA helix 44 studied by molecular dynamics simulations. Biopolymers 82: 504–520.

6. Banas P, Hollas D, Zgarbova M, Jurecka P, Orozco M, et al. (2010) Performance of molecular mechanics force fields for RNA simulations: Stability of UUCG and GNRA hairpins. J Chem Theory Comput 6: 3836-3849.

7. Wang J, Cieplak P, Kollman PA (2000) How well does a restrained electrostatic potential (RESP) model perform in calculating conformational energies of organic and biological molecules? J Comput Chem 21: 1049-1074.

8. Wilson DN, Nierhaus KH (2005) Ribosomal proteins in the spotlight. Crit Rev Biochem Mol Biol 40: 243–267.

9. Shandrick S, Zhao Q, Han Q, Ayida BK, Takahashi M, et al. (2004) Monitoring molecular recognition of the ribosomal decoding site. Angew Chem Int Ed Engl 43: 3177–3182.

10. Sanbonmatsu KY (2006) Energy landscape of the ribosomal decoding center. Biochimie 88: 1053–1059.

11. Borovinskaya MA, Pai RD, Zhang W, Schuwirth BS, Holton JM, et al. (2007) Structural basis for aminoglycoside inhibition of bacterial ribosome recycling. Nat Struct Mol Biol 14: 727–732.

12. Vaiana AC, Westhof E, Auffinger P (2006) A molecular dynamics simulation study of an aminoglycoside/A-site RNA complex: conformational and hydration patterns. Biochimie 88: 1061–1073.

13. Romanowska J, McCammon JA, Trylska J (2011) Understanding the origins of bacterial resistance to aminoglycosides through molecular dynamics mutational study of the ribosomal A-site. PLoS Comput Biol 7: e1002099.

14. Romanowska J, Setny P, Trylska J (2008) Molecular dynamics study of the ribosomal A-site. J Phys Chem B 112: 15227–15243.

15. Kaul M, Barbieri CM, Pilch DS (2006) Aminoglycoside-induced reduction in nucleotide mobility at the ribosomal RNA A-site as a potentially key determinant of antibacterial activity. J Am Chem Soc 128: 1261–1271.

16. Vicens Q, Westhof E (2001) Crystal structure of paromomycin docked into the eubacterial ribosomal decoding A site. Structure 9: 647–658.

17. Fourmy D, Recht MI, Blanchard SC, Puglisi JD (1996) Structure of the A site of Escherichia coli 16S ribosomal RNA complexed with an aminoglycoside antibiotic. Science 274: 1367–1371.

18. Fourmy D, Yoshizawa S, Puglisi JD (1998) Paromomycin binding induces a local conformational change in the A-site of 16 s rRNA. J Mol Biol 277: 333–345.

19. Ogle JM, Brodersen DE, Clemons WM, Tarry MJ, Carter AP, et al. (2001) Recognition of cognate transfer RNA by the 30S ribosomal subunit. Science 292: 897–902.

20. Barthel A, Zacharias M (2006) Conformational transitions in RNA single uridine and adenosine bulge structures: a molecular dynamics free energy simulation study. Biophys J 90: 2450–2462.

Table S1: **Deviations from the initial configuration during MD for the chosen subsystems:** averages of RMSD (± standard deviation, in Å), calculated for P and C_α atoms with respect to the initial structure. For each simulation variant the data from 3 MD trajectories are averaged over time.

	**all**		**h44**		**H69**		**S12**		**S12 (head)**		**S12 (tail)**	
**WT**	1.6	*(0.1)*	1.6	*(0.2)*	1.7	*(0.3)*	1.7	*(0.1)*	1.1	*(0.1)*	2.1	*(0.3)*
**K42A**	1.5	*(0.1)*	1.7	*(0.3)*	1.8	*(0.2)*	1.4	*(0.2)*	1.1	*(0.1)*	1.8	*(0.3)*
**R53A**	1.5	*(0.1)*	1.7	*(0.3)*	1.9	*(0.4)*	1.5	*(0.1)*	1.0	*(0.1)*	2.0	*(0.4)*
$\mathbf{WT_{PAR}}$	1.5	*(0.1)*	1.3	*(0.2)*	1.9	*(0.5)*	1.6	*(0.2)*	1.1	*(0.2)*	2.2	*(0.3)*
$\mathbf{K42A_{PAR}}$	1.4	*(0.1)*	1.4	*(0.1)*	1.9	*(0.4)*	1.3	*(0.1)*	1.0	*(0.1)*	1.7	*(0.3)*
$\mathbf{R53A_{PAR}}$	1.4	*(0.1)*	1.4	*(0.2)*	2.1	*(0.4)*	1.4	*(0.1)*	1.1	*(0.1)*	1.7	*(0.3)*

Table S2: **Duration of H-bonds created between N3 nitrogen of K43 and h44 nucleotides** (expressed as % of trajectory time). For each MD simulation run it is calculated as an average of occupancies for H-bonds of K43(N3) with 13 hydrogen acceptors: G1491 (O1P, O3'), A1492 (O1P, O2P, O2', O4', O5', N3, N7), A1493 (O1P, O2P, N1, N7). The data are also averaged over 3 trajectories for each variant (standard deviations in parentheses). One WT_{PAR} simulation is excluded from averaging (as in this case the K43····h44 interactions are not formed) and it is treated as an exception.

	**Duration of H-bonds (%)**				
	**#1**	**#2**	**#3**	**Average[#1,#2,#3]**	
**WT**	14.0	13.7	14.8	**14.2**	***(0.6)***
**K42A**	7.3	7.6	6.2	**7.0**	***(0.8)***
**R53A**	5.5	1.7	10.2	**5.8**	***(4.2)***
$\mathbf{WT_{PAR}}$	6.2	5.7	–	**5.9**	***(0.3)***
$\mathbf{K42A_{PAR}}$	7.9	8.5	4.5	**6.9**	***(2.2)***
$\mathbf{R53A_{PAR}}$	2.8	3.8	4.0	**3.5**	***(0.6)***

Table S3: **Percentage of time when H-bond between C1412(O2') and C910(O2') atoms is formed** during MD simulations (data are from three trajectories for each variant).

	**C1412···C910 (%)**
**WT**	19
**K42A**	24
**R53A**	21
$\mathbf{WT_{PAR}}$	6
$\mathbf{K42A_{PAR}}$	13
$\mathbf{R53A_{PAR}}$	8

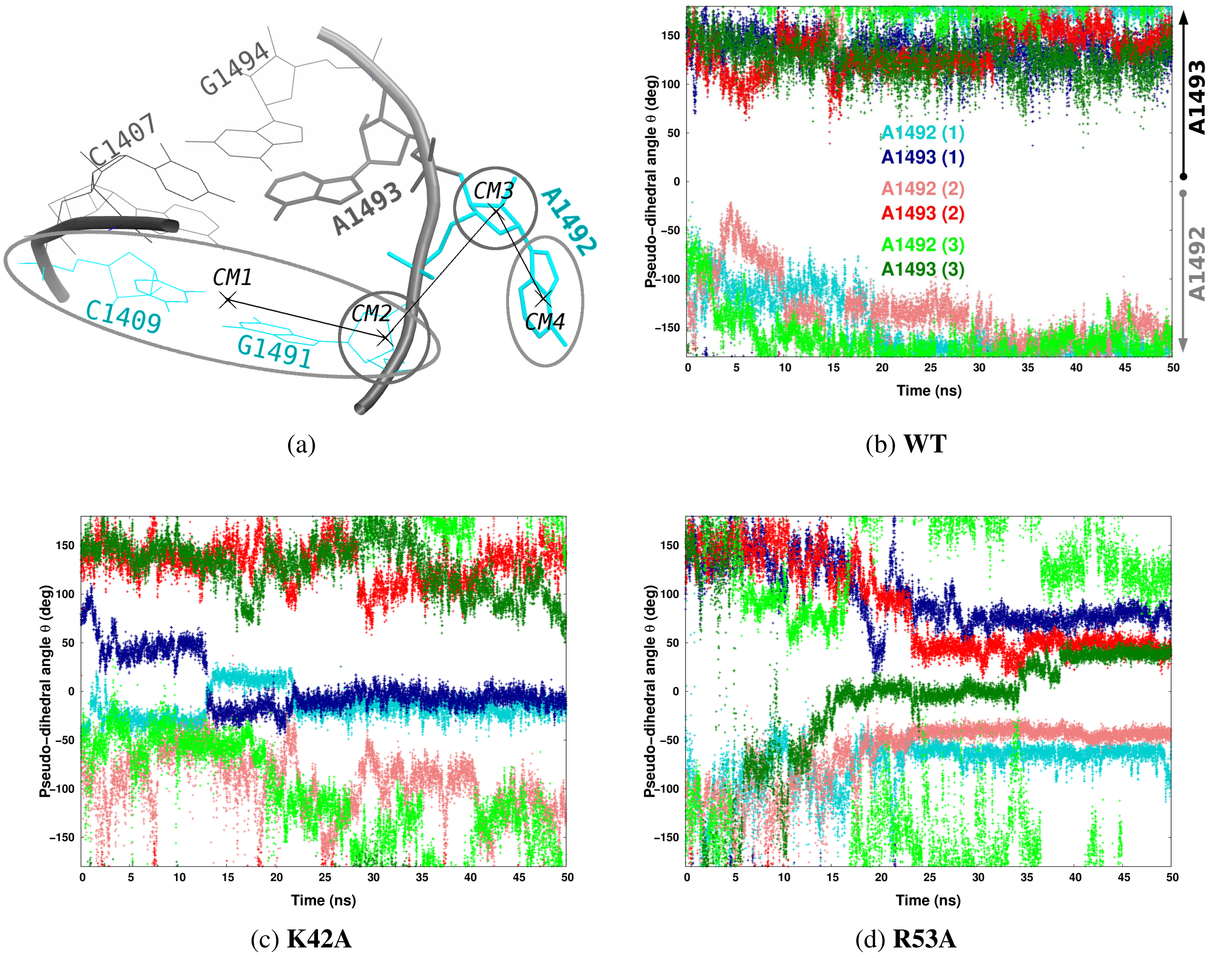

Figure S1: **Flipping of A1492 and A1493 (without the antibiotic) during MD simulations.** a) The θ pseudo-dihedral angle used to describe flipping (here shown for A1492) defined by the four pseudoatoms: CM1 – centre of mass of the neighboring base pair (C1409 and G1491), CM2 – centre of mass of the ribose of the neighboring nucleotide (G1491), CM3 – centre of mass of the flipping nucleotide ribose (A1492), CM4 – centre of mass of the flipping nucleobase (A1492). The values of θ for A1492 and A1493 versus time in the variants: b) WT, c) K42A and d) R53A in each MD trajectory (the simulation numbers are shown in parentheses in [b]). The typical direction of A1492 and A1493 flipping movement during mRNA decoding process (through the minor groove of the rRNA helix) is indicated by arrows in panel (b).

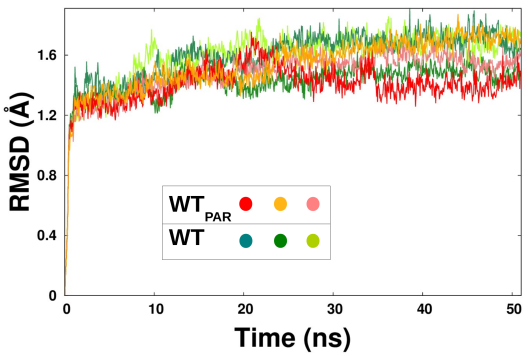

(a) **Full system**

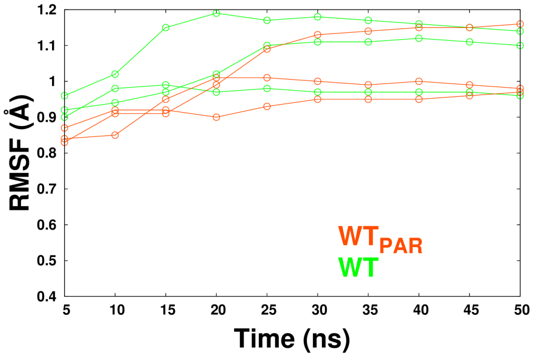

(b) **h44 helix**

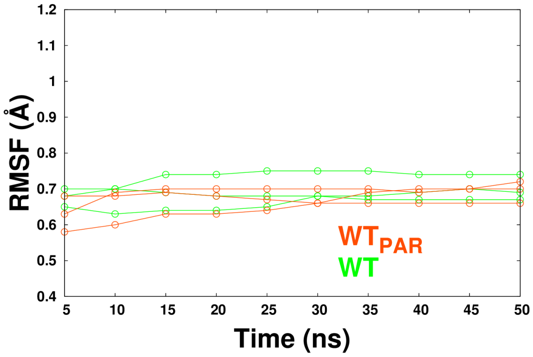

(c) **S12 protein – 'head'**

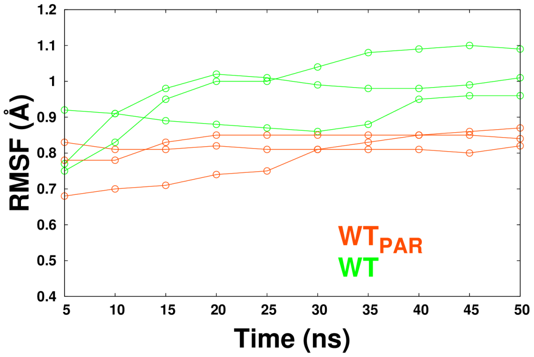

(d) **H69 helix**

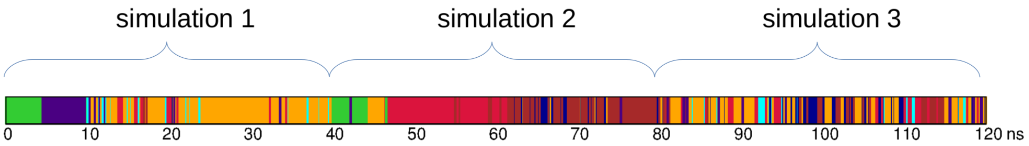

(e) **membership of mutual A1492/A1493 conformations in clusters**

Figure S2: **Convergence of MD simulations for the wild-type systems with and without paromomycin.** a) Evolution of RMSD from the starting structures for the solute heavy atoms in each of the 3 simulations in WT and WT_{PAR} variants. b)-d) Cumulative RMS fluctuations. The average fluctuations were calculated for the selected subsets of residues as a function of time: b) the h44 helix, c) 'head' of the S12 protein and d) the H69 helix. Panel (e) shows the membership of mutual A1492 and A1493 conformations in time to different clusters in three WT simulations (without paromomycin). Different colors mark different clusters. Clustering was done for conformations gathered from all 3 trajectories, with 3.5 Å radius (see 'Methods' in the main text).

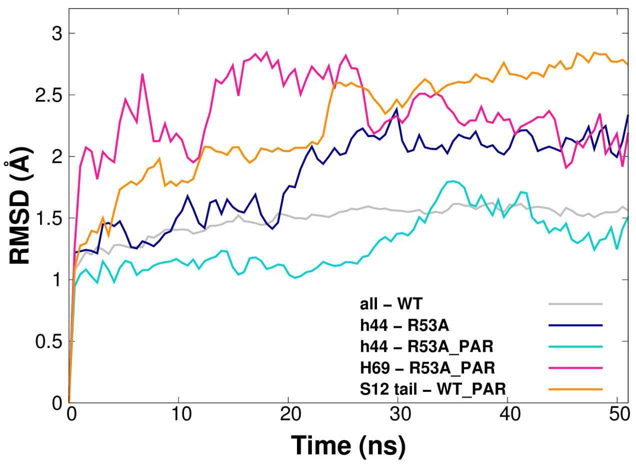

(a)

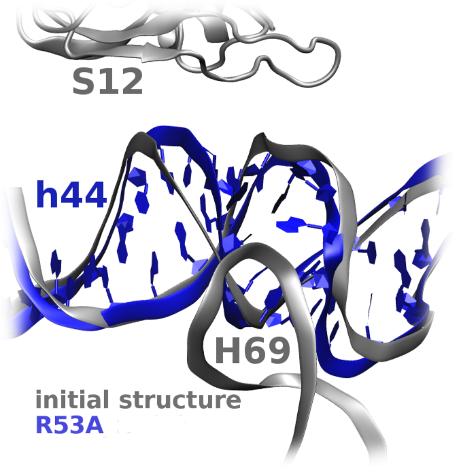

(b)

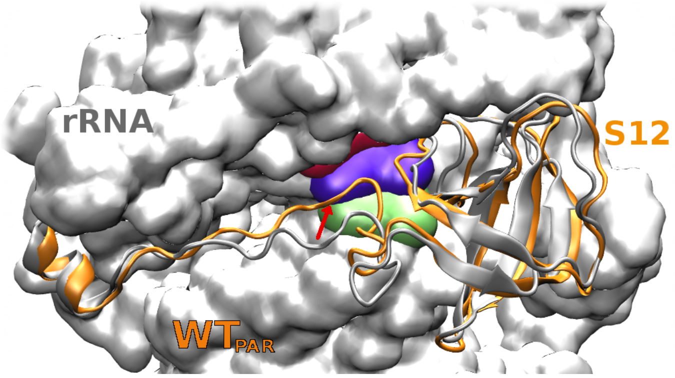

(c)

Figure S3: **Structural stability in the simulated systems.** a) RMSD (P/Cα) versus time for h44 and H69 helices and the "tail" of the S12 protein in the chosen MD simulations. The structures with the highest RMSD in the trajectories selected from those presented in (a): b) h44 and c) S12 and the three nucleotides mentioned in the text: U911 – in light green, C912 – blue, A913 – red; red arrow indicates the direction of conformational change from the initial structure.

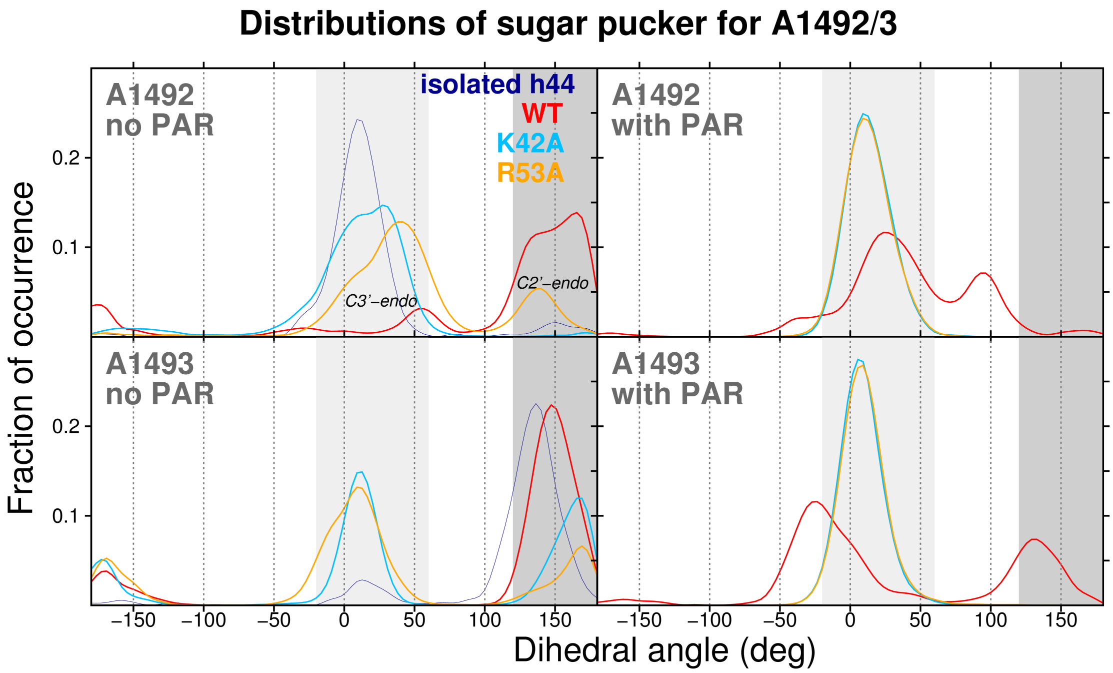

Figure S4: **Distributions of sugar pucker phase for A1492 and A1493 in MD simulations.** The regions corresponding to C2'-*endo* and C3'-*endo* configurations are shaded. The data are cumulative from three independent MD trajectories per simulation variant.

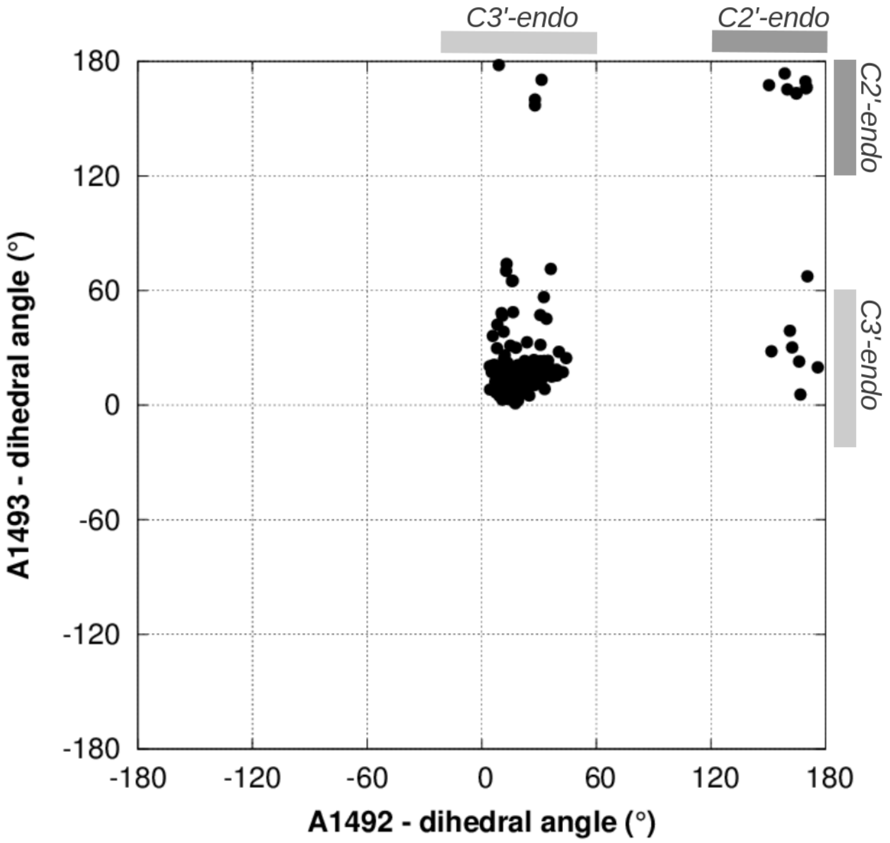

Figure S5: **Sugar pucker phase of A1492 vs A1493 gathered from 170 crystal structures of the bacterial ribosome.** The C2’-*endo* and C3’-*endo* ranges are indicated.

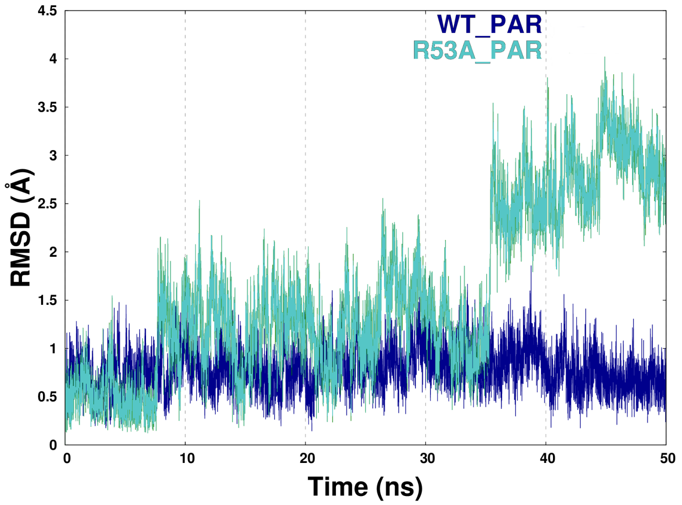

(a)

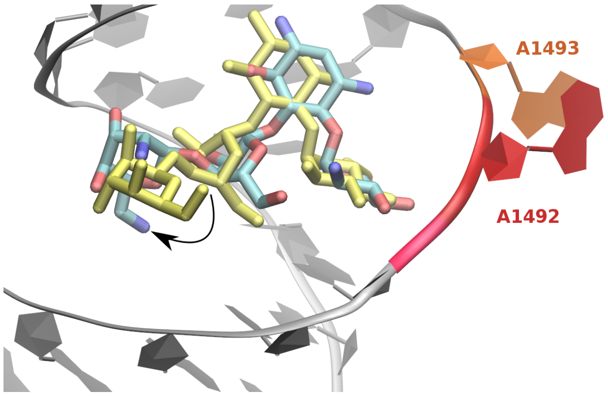

(b)

Figure S6: **Structural stability of paromomycin in MD simulations:** a) RMSD in time for the ring IV of paromomycin (see Fig 1e, main text) in the two simulations: one of WT_{PAR} with stable PAR and the other of $R53A_{PAR}$ variant, with exceptionally unstable antibiotic. b) Conformational change of paromomycin in the $R53A_{PAR}$ trajectory: the initial structure – in yellow, the conformation with the highest RMSD – colored by atom type.

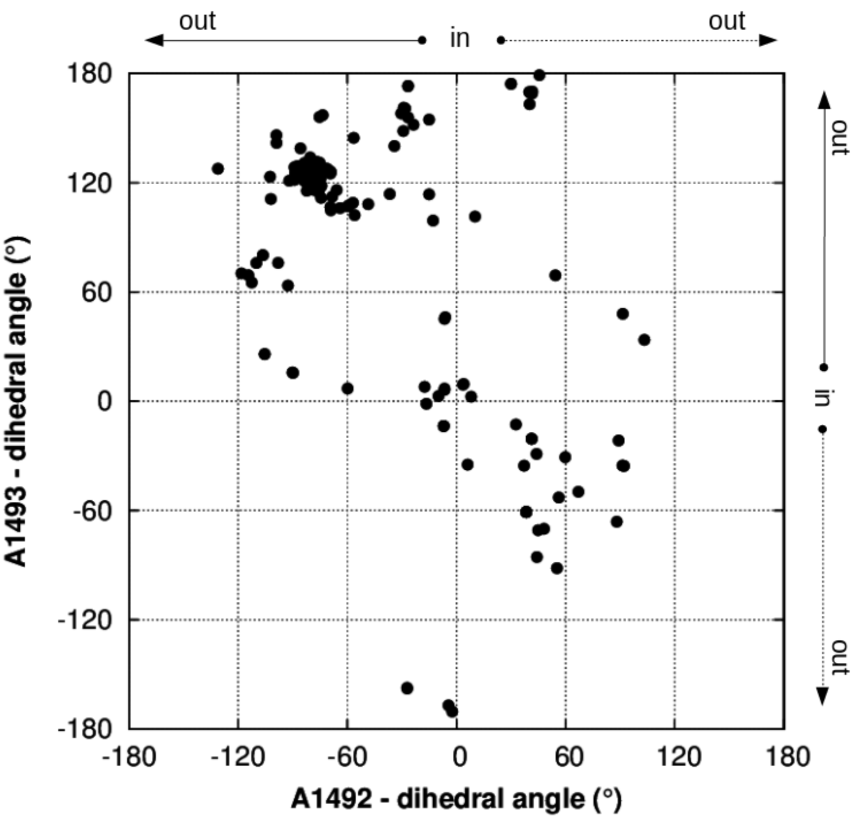

Figure S7: **Positions of A1492 vs A1493 in crystal structures of the bacterial ribosomes.** The plots of C1'(C1409)-C1'(G1491)-C1'(A1492)-N9(A1492) versus C1'(C1407)-C1'(G1494)-C1'(A1493)-N9(A1493) dihedral angles. These angles were shown to characterize well flipping of the nucleotides in bulges (20) and they are analogical to the θ angles defined in Fig S1a. The values close to 0 correspond with the flipped-in conformations, while the values close to ± 180 – the flipped-out states. The arrows indicate the two directions of flipping-out: through the minor and major groove (marked by continuous and dotted lines, respectively). Data are gathered from 167 structures.

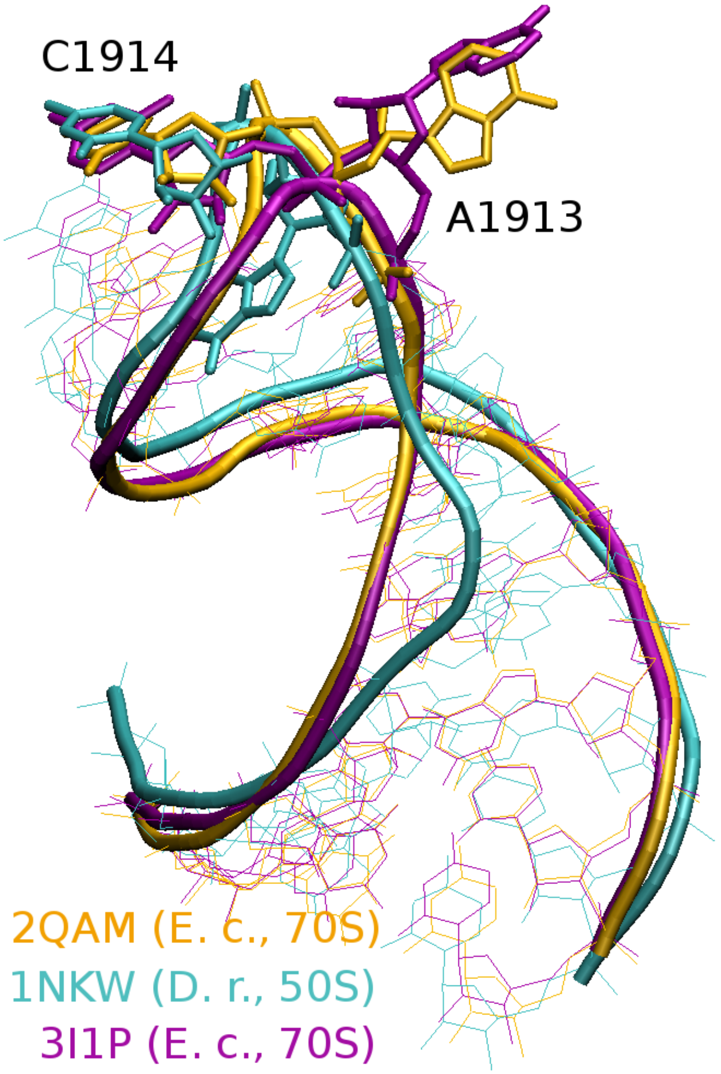

Figure S8: **Comparison of H69 conformations in different crystal structures of bacterial ribosomes.** Abbreviations: E. c. – *E. coli*, D. r. – *D. radiodurans*. PDB codes are given.
